# Ectopic RING zinc finger gene from hot pepper induces totally different genes in lettuce and tobacco

**DOI:** 10.1007/s11032-018-0812-3

**Published:** 2018-05-16

**Authors:** Mahipal Singh Kesawat, Dong Kyun Kim, Naheed Zeba, Mi Chung Suh, Xinli Xia, Choo Bong Hong

**Affiliations:** 1 School of Biological Sciences, Institute of Molecular Biology and Genetics, Seoul National University, Seoul, 151-742 South Korea; 2 Present Address: Department of Genetics and Plant Breeding, Sher-e-Bangla Agricultural University, Dhaka, 1207 Bangladesh; 3 Present Address: Department of Bioenergy Science and Technology, Chonnam National University, Gwangju, 500-757 South Korea; 4 College of Biological Sciences and Technology, Beijing Forestry University, Beijing, 100083 People’s Republic of China

**Keywords:** Ectopic expression, RING zinc finger, Lettuce, Tobacco, Hot pepper, Gene expression pattern

## Abstract

Advances in molecular biology have improved crops through transferring genes from one organism to new hosts, and these efforts have raised concerns about potential unexpected outcomes. Here, we provide evidence that a gene with a specific function in one organism can yield completely different effects in a new host. *CaRZFP1* is a C3HC4-type RING zinc finger protein gene previously isolated from a cDNA library for heat-stressed hot pepper. In our previous work investigating in vivo *CaRZFP1* function, we transferred *CaRZFP1* into tobacco; transgenic tobacco exhibited enhanced growth and tolerance to abiotic stresses. As further analysis of *CaRZFP1* ectopic expression in a heterologous host plant, here we mobilized and constitutively overexpressed *CaRZFP1* in lettuce. In contrast to tobacco, transgenic lettuce exhibited poorer growth and delayed flowering compared with vector-only controls. To identify genes that might be involved in this phenotypic effect, transcriptome analyses on transgenic plants of both species were performed, uncovering dozens of genes that reflect the different outcomes between tobacco and lettuce. These included protein kinase, transcriptional factor, transporter protein, hormone and metabolism-related genes, and some unannotated genes. The opposite effects of *CaRZFP1* ectopic expression in lettuce and tobacco address concerns of unexpectedly different outcomes in different host species.

**Electronic supplementary material:**

The online version of this article (10.1007/s11032-018-0812-3) contains supplementary material, which is available to authorized users.

## Introduction

Advances in molecular biology have improved crops through transferring genes from one organism to new hosts. Studies showed that GM crops have increased crop yields by 21% and reduced pesticide usage by 37%. The agronomic and economic benefits of GM crops are apparently large and significant (Klumper and Qaim 2014). Until now, US-based companies have been only developers of GM crops which cultivated on a large scale as authorized. Other countries are now producing GM crops with little or no information systematically provided. The US regulatory authorities are beginning to accept the possible need of European style GM crop regulations to mitigate or avoid future risks to the environment and human health. A group of scientists did an extensive review of research on the safety of GM crops over the past years. They found that the scientific research conducted so far has not documented any significant adverse effects directly associated with the use of GM crops on human health and environment (Nicolia et al. 2014).

The zinc finger domain in zinc finger proteins (ZFPs) consists of 20–100 amino acid residues and is found ubiquitously from prokaryotes to eukaryotes (Krishna et al. 2003). Cysteine and histidine residues in the domain stabilize the structure through binding to one or more zinc ions (Laity et al. 2001). Comprising a large and diverse gene family, ZFPs have considerable variation in structure and recognition sequences that interact with DNA, chromatin, RNA, and proteins. Thus, ZFPs are linked to major, diverse biological functions, including transcriptional activation, DNA recombination, translational processes, signal transduction, programmed cell death, membrane association, as well as protein folding and assembly (Gamsjaeger et al. 2007; Zhang et al. 2013). ZFPs participate in various fundamental aspects of plant growth and development, such as phytohormone response, signal transduction, and responses to abiotic or biotic stimuli (Chai et al. 2015; Larrieu and Vernoux 2009; Liu et al. 2016). Originally, zinc fingers were classified based on the number and order of cysteine and histidine residues, resulting in Cys_2_His_2_, Cys_4_, Cys_6_, and other similarly named types. Recent ZFP groupings instead use unique characteristics of the folded domain from the protein backbone. The most common zinc finger “fold groups” are Cys_2_His_2_-like (classic zinc finger), treble clef, and zinc ribbon. Most of the Cys_2_His_2_-like ZFPs are also commonly called “Really Interesting New Gene (RING)” finger proteins. They have been further grouped into several classes (C2H2, C2C2, C2C2C2C2, C2HCC2C2, C2HC, C2HC5, C3HC4, C3H2C3, C3H, C3HDC3, C3HGC3, C3H2SC2, C4HC3, C4, C4C4, C5HC2, C6, and C8) according to the number of conserved cysteine and histidine residues, the spacing between them, and their specific molecular functions (Laity et al. 2001; Krishna et al. 2003; Gamsjaeger et al. 2007).

Previously, we isolated a C3HC4-type RING zinc finger protein gene, *CaRZFP1*, from a cDNA library of heat-stressed hot pepper (*Capsicum annuum*). Various environmental stresses (e.g., heat, cold, dehydration, and high salinity) induce *CaRZFP1* transcription in hot pepper. When the *CaRZFP1* open reading frame (ORF) was transformed into tobacco (*Nicotiana tabacum*) and ectopically expressed, growth and tolerance to abiotic stresses were enhanced in the transgenic tobacco plants (Zeba et al. 2009). In this study, we mobilized and expressed *CaRZFP1* in lettuce (*Lactuca sativa*) to further analyze the effect of *CaRZFP1* ectopic expression in a heterologous host plant. Contrasting with tobacco, transgenic lettuce exhibited poorer growth than vector-only controls, specifically delayed flowering, weakened leaf growth, shorter plant height, and stunted root growth. This report examines possible mechanisms underlying the different effects caused by the same RING zinc finger protein gene expressed in transgenic lettuce versus transgenic tobacco.

## Materials and methods

### Plant material and growth condition

Seeds of hot pepper (*C. annuum* L. cv. Bu Gang), tobacco (*N. tabacum* L. cv. Wisconsin 38), and lettuce (*L. sativa* L. cv. Chung Chima) were sown on soil in plastic pots and reared in a growth chamber under controlled conditions of 25 °C, 60% relative humidity, and a 16-h photoperiod from white fluorescent lamps (200 μmol photons m^−2^ s^−1^). Vector-only or *CaRZFP1-*transgenic tobacco and lettuce plants were further grown in a greenhouse insulated with a dual door at 25 ± 2 °C under natural lighting with additional fluorescent lighting to maintain a 16-h photoperiod. Transgenic tobacco and lettuce plants carrying recombinant expression construct of *CaRZFP1* and the vector-only were self-fertilized, and T_1_ generation seeds were harvested. T_1_ generation seeds with the transgene expressed were selected on a kanamycin-containing medium, raised for flowering, and self-fertilized again to obtain T_2_ generation seeds. This selection, self-fertilization, and rearing in a greenhouse was repeated to get further generations, up to T_5_ generation. For the growth assay, transgenic lines were transferred to soil in plastic pots and reared in a growth chamber as described above. Four- to 5-week-old plants were used for nucleic acid extraction.

### Generation of transgenic lettuce plants overexpressing *CaRFZP1*

The open reading frame of *CaRZFP1* was amplified by PCR with a primer pair covering both termini. The 5′ primer was 5′-ATATGGATCCATGCAGAAGTCAACTGCTACG-3′ and the 3′ primer was 5′-ATATGGATCCCTAACCAAACAAATATAGGAATAC-3′ with the underlined *Bam*HI restriction site. PCR was carried out with the initial reaction of 94 °C for 5 min; followed by 30 cycles of 94 °C for 1 min, 55 °C for 30 s, and 72 °C for 1 min; with a final reaction of 10 min at 72 °C. The amplified PCR product was then digested with *Bam*HI and ligated into the pBKS1-1 plant expression vector (Suh et al. 1994) at the *Bam*HI site to locate the open reading frame under the control of the CaMV35S promoter. Nucleotide sequence of the cloned coding region in pBKS1-1 was confirmed by an automated DNA sequencer (3730xI DNA Analyzer, Applied Biosystems). The *CaRZFP*-pBKS1-1 plasmid was electroporated into the *Agrobacterium tumefaciens* strain LBA4404 and used for transformation of lettuce. In brief, sterilized hypocotyls of lettuce were cut to 1–2 cm length and infected with the *Agrobacterium* cells carrying the expression construct. After co-cultivation for 24 h, explants were washed with sterilized MS medium and placed in a shoot induction medium containing 200 mg/ml kanamycin and 100 mg/ml cefotaxime. Kanamycin-resistant shoots were selected and transferred to a root induction medium containing 200 mg/ml kanamycin (Horsch et al. 1985). The putative *CaRZFP1*-transgenic plants were then transferred to soil and reared in a growth chamber, then in a greenhouse as described above.

### RNA and DNA blot analyses

Total RNA was extracted from plant tissues frozen in liquid nitrogen. Briefly, frozen tissue was ground to powder, homogenized in 3 ml of extraction buffer (100 mM LiCl, 100 mM Tris-Cl pH 8.0, 10 mM EDTA, and 1% SDS), a mixture of 3 ml of chloroform-isoamyl alcohol (24:1) was added, followed by vortexing and centrifugation at 10,000×*g* for 25 min at 4 °C. The supernatant was transferred to a 1.5-ml microcentrifuge tube, extraction was repeated with 1.5 ml chloroform-isoamyl alcohol (24:1) mixture, and precipitated in an equal volume of 4 M LiCl at − 70 °C for 2 h. After centrifugation, the pellet was washed with cold 70% ethanol and dissolved in DEPC-treated distilled water. Twenty micrograms of total RNA, for each sample, was loaded onto a 1.2% agarose gel with formaldehyde. To check the integrity of the sample, RNA was visualized by staining with ethidium bromide and UV illumination after electrophoresis, and RNA was transferred onto nylon membranes (Hybond-N^+^, GE Healthcare Bio-Sciences), followed by cross-linking with UV illumination. To generate *CaRZFP1*-specific probe, the coding sequence of *CaRZFP1* was amplified by PCR and labeled with α^32^P-dCTP. Pre-hybridization for 2 h and hybridization for 16 to 22 h were done in a solution of 1 M dibasic sodium phosphate (pH 7.2), 14% SDS, and 20 μl of 1 mM EDTA (pH 8.0) at 65 °C. For RNA blot analysis using oligonucleotides, 60 mer oligonucleotides were end labeled with γ^32^P-dATP and polynucleotide kinase. The hybridization procedure was the same as the PCR-generated probe, except the hybridization and washing temperature which was at 58 °C. After hybridization, the membrane was washed twice with 1X SSPE and 0.1% SDS for 15 min, once at room temperature, then at 65 °C, and the membrane was washed several times in 0.5X SSPE and 0.1% SDS at 65 °C (Sambrook and Russell 2001). The blots were exposed to a phosphoimager screen and an image was developed in a phosphoimager (Typhoon 8600, Molecular Dynamics). For RT-PCR analysis, total RNA preparation was predigested with DNase I (Takara Bio.) at 37 °C for 30 min, and the first cDNA strand was generated by reverse transcribing RNA using AMV reverse transcriptase (Promega) at 42 °C for 1 h. cDNA was quantified using a spectrophotometer (ND-1000, NanoDrop Technologies) and subjected to PCR using a primer pair of 5′-ATGCAGAAGTCAACTGCTACG-3′ and 5′-CTAACCAAACAAATATAGGAATAC-3′ covering the open reading frame of *CaRZFP1* in a PCR mixer (Promega) on a DNA thermal cycler (MJ Mini thermal cycler, Bio-Rad) with a following profile: initial step at 94 °C for 5 min followed by 30 cycles of 94 °C for 1 min, 55 °C for 30 s, and 72 °C for 1 min and a final step at 72 °C for 10 min. Amplified RT-PCR products were confirmed by DNA nucleotide sequencing. All chemicals used were from Sigma-Aldrich, Becton, Dickinson and Co. and Duchefa Biochemie, otherwise mentioned.

### Phenotypic assay

Seeds of *CaRZFP1-*transgenic and vector-only lettuce plants were sown in plastic pots and reared in a growth chamber or a greenhouse under the controlled conditions as described above for the phenotypic examination during the vegetative growth. The plants were moved to a greenhouse under the controlled conditions as described above to follow the full life cycle. Evaluated parameters were leaf length, leaf width, leaf fresh weight, root mass, root fresh weight, plant height, flowering time, flower size, and seed morphology.

### Histological and in situ hybridization analyses

To examine the effect of ectopic expression of *CaRZFP1* at the cellular level, the *CaRZFP1-*transgenic and the vector-only lettuce and tobacco (Zeba et al. 2009) plants were anatomically assayed. Leaves, stems, and roots of the plants were taken and placed them immediately in 4% paraformaldehyde and vacuum infiltrated until the tissues sank. The samples were washed twice with 1X PBS (130 mM NaCl, 7 mM Na_2_HPO_4_, and 3 mM NaH_2_PO_4_, pH 7.0) for 30 min each and dehydrated using increasing concentrations of ethanol series (10, 30, 40, 50, 60, 70, and 85%), 60 min for each step. The samples were then incubated in 95% ethanol plus 0.1% eosin-Y overnight. Next day, samples were incubated in 100% ethanol, histoclear/ethanol series of 25, 50, and 75%, and twice in 100% histoclear, 60 min for each step. Histoclear was replaced with paraplast and kept the samples at 60 °C overnight. Paraplast was changed three times in a day with fresh molten wax and cool to room temperature to solidify. Samples were cross-sectioned in 6- to 8-μm thickness using a microtome (Microm HM340E, Microm International GmbH). The cross sections were floated on DEPC-treated water and dried overnight at 40 °C to fix the sections onto Superfrost Plus Microscope Slides (Thermo Fisher Scientific). The cross sections were deparaffinized by dipping the slides in xylene for 10 min and rehydrated with a graded ethanol series (100, 95, 90, 80, 60, and 30%). After rinsing with distilled water, the sections were stained with 1% safranin and observed under light microscopes (Leica DC500, Leica Microsystems; Olympus BX51, Olympus Corporation). In situ hybridization to visualize *CaRZFP1* transcript was carried out as described by Brewer et al. 2006. Briefly, the cross sections were fixed onto Superfrost Plus Microscope Slides and deparaffinized. The sections were incubated in 1X PBS for 5 min, permeabilized by proteinase K treatment for 30 min, fixed in 4% paraformaldehyde in phosphate buffer (pH 7.0) for 10 min, acetylated twice with 0.5% acetic anhydride in 0.1 M triethanolamine for 10 min each, and the slides were washed in 1X PBS for 5 min. Then, the sections were hybridized with in vitro transcribed digoxigenin-labeled *CaRZFP1* sense or antisense riboprobes which were synthesized from linearized pBluescript plasmids containing *CaRZFP1* as described in the (SP6/T7) DIG RNA labelling Kit (Roche Applied Science). Briefly, the root sections were hybridized with equal concentrations of either sense or antisense RNA probes in a hybridization buffer [50X Denhardt’s, 50% dextran sulfate, 100 mg/ml tRNA, 50% formamide, 10X in situ hybridization salts (3 M NaCl, 100 mM Tris-HCl pH 8.0, 100 mM Na-phosphate pH 6.8, and 50 mM EDTA pH 8.0)], covered with parafilm, and incubated at 53 °C overnight in a humidity chamber. After hybridization, the slides were put into 2X SSC for 60 min to allow parafilm to float off. Then, the slides were washed twice in 0.2X SSC at 55 °C for 1 h each and equilibrated twice in 1X NTE (2.5 M NaCl, 50 mM Tris-Cl pH 8.0, and 5 mM EDTA pH 8.0) at 37 °C with gentle agitation for 5 min each. The slides were then put into 1X NTE buffer with 20 μg/ml RNAse A for 30 min at 37 °C with gentle agitation, rinsed twice in 1X NTE for 2 min each, washed in 0.2X SSC at 55 °C for 1 h, equilibrated in 1X PBS for 5 min at room temperature, incubated in blocking solution [1% blocking reagent (Roche Diagnostics Gmbh), 100 mM Tris-Cl pH 7.5, and 150 mM NaCl] for 45 min at room temperature, and washed with a washing buffer (1% BSA, 100 mM Tris-Cl pH 7.5, 150 mM NaCl, and 0.3% Triton X-100) for 45 min. The anti-digoxigenin antibody (Roche Applied Science) was diluted to 1:1250 ratio in the washing buffer, and 200 μl of anti-digoxigenin antibody solution was directly applied onto the slide carrying the parafilm strips. The slides were incubated in the dark for 2–3 h at room temperature or overnight at 4 °C, washed four times in washing buffer for 15 min each on a shaking platform, and the slides were transferred into 1X PBS, incubated for 2 min, and equilibrated in TN buffer (100 mM Tris-HCl pH 9.5 and 100 mM NaCl) twice for 2 min each. Staining solution was prepared immediately before use by adding 20 μl nitro-blue tetrazolium/5-bromo-4-chloro-3′-indolyphosphate per 1 ml TN buffer. The slides were covered with the staining solution and kept in a plastic box in the dark at room temperature for 2–3 days. Development of staining was monitored under a light microscope, and once the color reaction was complete, the slides were placed in TE buffer for 5–10 min to stop the staining reaction. The slides were washed in 1X PBS for 5 min, and images were captured with a light microscope (Olympus BX51).

### Transcriptome analysis

Four T_4_ generation *CaRZFP1*-transgenic lettuce lines (no. 6, no. 12, no. 14, and no. 16) and three vector-only control lettuce lines (V10, V30, and V38) were selected for transcriptome analysis using *Arabidopsis* 44K oligo microarray (Agilent Technology) to maintain the methods used for the transcriptome profiling of *CaRZFP1-*transgenic tobacco (Zeba et al. 2009). Total RNA was extracted from 4-week-old plants, and genomic DNA was removed by DNase I (Takara Bio Inc.) digestion. Synthesis of cRNA probes and hybridization were carried out using Agilent’s low RNA Input linear amplification kit (Agilent Technologies) according to the manufacturer’s instructions. Briefly, total RNA 1 μg was mixed with T7 promoter primer mix and incubated at 65 °C for 10 min, cDNA master mix (5X first strand buffer, 0.1 M DTT, 10 mM dNTP mix, RNase-Out, and MMLV-RT) was added, incubated at 40 °C for 2 h, and reverse transcription and dsDNA synthesis were terminated by incubating at 65 °C for 15 min. Transcription of the dsDNA was done by adding transcription master mix (4x transcription buffer, 0.1 M DTT, NTP mix, 50% PEG, RNase-Out, inorganic pyrophosphatase, T7-RNA polymerase, and cyanine 3-CTP) and incubating at 40 °C for 2 h. The labeled cRNA was purified on cRNA cleanup module and hybridized to the microarrays at 65 °C for 17 h. After hybridization, microarrays were washed for 1 min at room temperature with GE Wash Buffer 1, again for 1 min at 37 °C with GE Wash buffer 2, and dried immediately by centrifugation at 400×*g* for 2 min at room temperature. The hybridization images were scanned using a DNA microarray scanner and quantified with the feature extraction software 9.3.2.1 (Agilent Technologies). Data normalization and calculation of fold change were performed using GeneSpringGX 7.3 (Agilent Technologies). Greater than twofold changes with *p* < 0.05 were set as the threshold for statistical significance (McCarthy and Smyth 2009).

#### Data availability statement

All the data in this publication will be available upon request.

## Results

### *CaRZFP1* overexpressing transgenic lettuce plants showed hampered growth and development

**Fig. 1:**
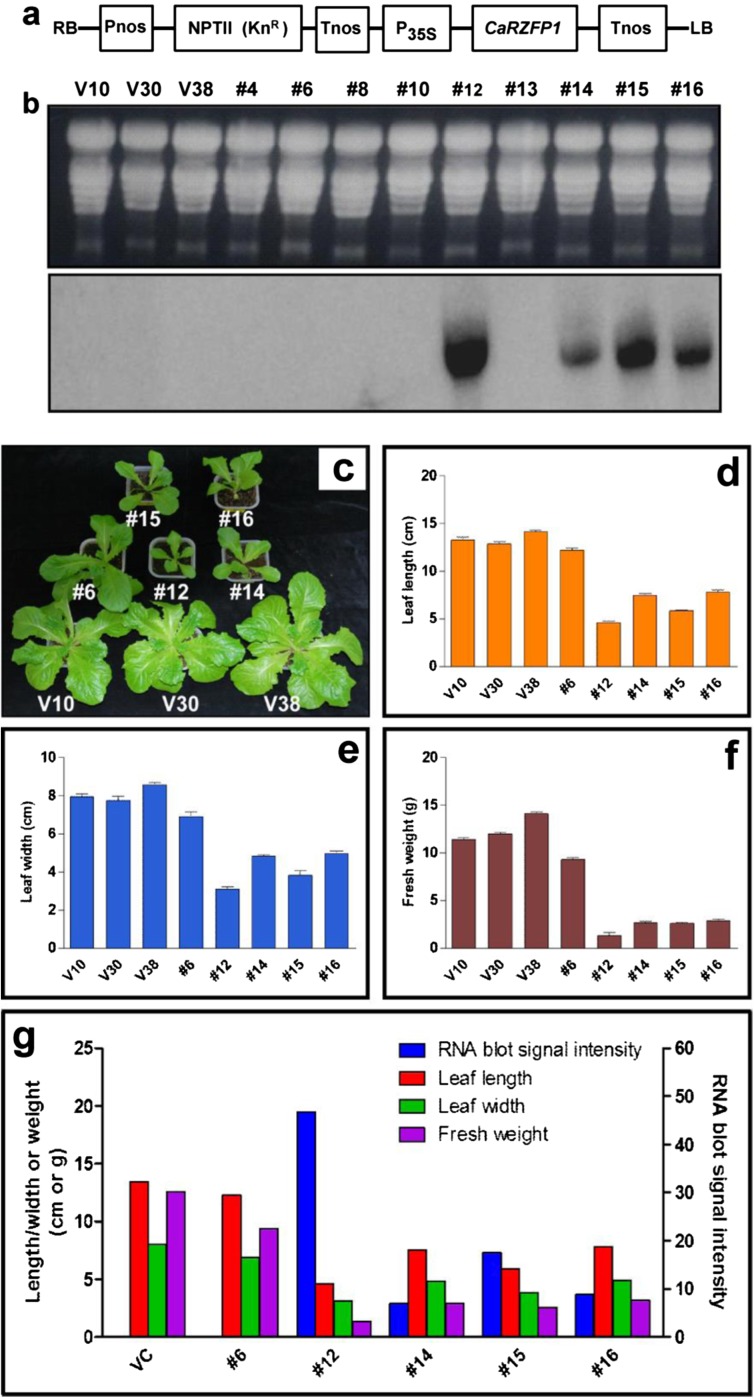

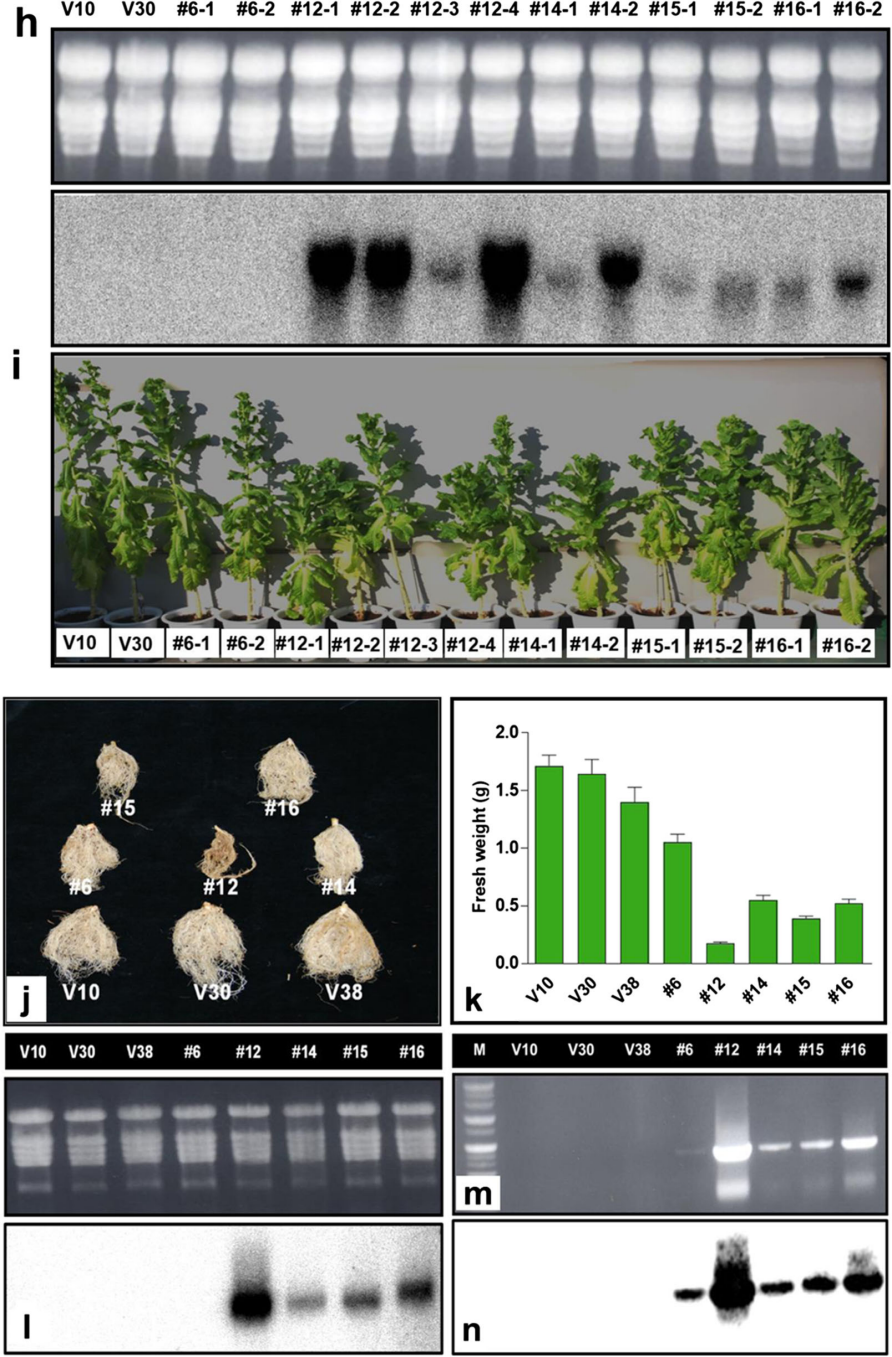
*CaRZFP1* overexpressing transgenic lettuce plants showed hampered growth and development. **a** Diagrammatic representation of pBKS1-1-*CaRZFP1* construct used for lettuce transformation. For the expression vector pBKS1-1, only the region inside of the border sequences, RB and LB, that was actually transferred into the lettuce genome is shown. **b** RNA blot hybridization results for vector-only and putative T_1_ transgenic plant lines. Total RNA was separated by electrophoresis on a 1.2% formaldehyde agarose gel and blotted to a Hybond-N nylon membrane. Separated RNA was stained with ethidium bromide for visualization with UV illumination. The blots were hybridized to ^32^P-labeled *CaRZFP1* probe. **c** Typical examples of transgenic lettuce plant lines no. 6, no. 12, no. 14, no. 15, and no. 16 and lettuce plants carrying only the vector after 4 weeks since seed imbibition. **d** Comparison of leaf length of the plants in **c**. **e** Comparison of leaf width of the plants in **c**. **f** Comparison of fresh weight of the plants in **c**. **g** The data in **d**, **e** and **f** were aligned with *CaRZFP1* transcript level analyzed by RNA blot hybridization. V10, V30 and V38, lettuce plants carrying only the expression vector. Error bars show standard deviation. VC, average of V10, V30 and V38. **h** RNA blot hybridization results of vector-only or putative T_3_ *CaRZFP1*-transgenic lettuce lines that are shown in **i**. **i** Typical examples of T_3_ transgenic lettuce plant lines no. 6, no. 12, no. 14, no. 15 and no. 16 and lettuce plants carrying only the vector after 12 weeks since seed imbibition. **j** Typical roots of T_3_ transgenic lettuce plant lines no. 6, no. 12, no. 14, no. 15 and no. 16 and lettuce plants carrying only the vector after 4 weeks since seed imbibition. **k** Comparison of root mass of the plants in **j**. **l** RNA blot hybridization results for the plants in **j**. **m** RT-PCR results for the same samples in **l**. **n** RT-PCR results in **m** was further confirmed by DNA blot analysis with ^32^P-labeled *CaRZFP1* probe. M, size marker. Error bars show standard deviation

To investigate the effect of *CaRZFP1*-ectopic expression, transgenic lettuce was generated that overexpressed *CaRZFP1* under the control of cauliflower mosaic virus 35S promoter (Fig. 1a). This construct and the vector without *CaRZFP1* were mobilized into the lettuce genome. The putative *CaRZFP1*-transgenic lettuce plants were selected on kanamycin-containing medium and planted in soil. Next, T_1_ generation lines were screened with RNA blot analysis to evaluate *CaRZFP1* transcript levels and confirm transgenicity (Fig. 1b). In the representative examples shown, T_1_ transgenic line no. 12, no. 14, no. 15, and no. 16 exhibited different but significant levels of ectopically expressed *CaRZFP1* transcript under normal growth conditions. Line no. 6 expressed very low *CaRZFP1* levels undetectable via RNA blot hybridization; transcripts were thus confirmed with RT-PCR and DNA blot analyses (Fig. S1). Confirmed T_1_ lines were reared in a greenhouse and repeatedly self-fertilized to obtain next-generation seeds (T_2_ to T_5_). Transgenicity of all putative transgenic plants from different generations was confirmed with RNA blot analysis. We noticed that most *CaRZFP1*-transgenic lettuce plants with detectable *CaRZFP1* transcript levels exhibited growth impairment, more strongly in some lines than in others. Starting from T_2_, we categorized transgenic lettuce lines according to *CaRZFP1* transcript expression levels (low, medium, and high). Five independent T_2_ lines were selected based on *CaRZFP1* transcript levels and denoted with numerals 6, 12, 14, 15, and 16. Additionally, three vector-only lines (V10, V30, and V38) with morphology typical of nontransgenic lettuce were also selected for further analyses (Fig. 1b).

A strong negative correlation existed between *CaRZFP1* transcript level and growth robustness in the vegetative stage, i.e., stronger *CaRZFP1* expression, was tightly linked to severe growth retardation. Growth and development retardation was noticeable soon after germination and continued thereafter; transgenic lettuce exhibited poorer overall growth compared with vector-only plants, including diminishing leaf growth, shorter plant height, and lower fresh weight (Fig. 1c–f). Line no. 12 exhibited the highest *CaRZFP1* expression and the most impeded growth among all transgenic lettuce lines. Line no. 14, no. 15, and no. 16 were more moderate in terms of both *CaRZFP1* expression and hampered growth. Line no. 6’s very low, near-undetectable *CaRZFP1* expression corresponded to a negligible effect on growth and development; its phenotype was similar to vector-only controls. Ectopic *CaRZFP1* expression thus caused pleiotropic developmental changes. After 28 days of germination, the average leaf lengths of *CaRZFP1*-transgenic lettuce line no. 6, no. 12, no. 14, no. 15, and no. 16 were 13.8 ± 0.47, 9.3 ± 0.19, 11.0 ± 0.62, 10.4 ± 0.74, and 11.5 ± 0.89 cm, while leaves from vector-only V10, V30, and V38 averaged 15.4 ± 0.53, 14.4 ± 0.30, and 14.5 ± 0.32 cm (Fig. 1d). Average leaf widths of no. 6, no. 12, no. 14, no. 15, and no. 16 were 6.9 ± 0.41, 4.0 ± 0.22, 5.6 ± 0.78, 5.1 ± 0.24, and 5.0 ± 0.74 cm, whereas V10, V30, and V38 leaf widths averaged 9.0 ± 0.25, 8.4 ± 0.31, and 8.6 ± 0.29 cm (Fig. 1e). Average fresh weights of no. 6, no. 12, no. 14, no. 15, and no. 16 were 4.4 ± 0.23, 1.3 ± 0.34, 2.9 ± 0.15, 2.7 ± 0.21, and 3.4 ± 0.24 g, while those of V10, V30, and V38 were 6.6 ± 0.43, 5.6 ± 0.34, and 5.7 ± 0.20 g (Fig. 1f). The correlation between *CaRZFP1* transcript level and a negative phenotypic effect was repeatedly maintained through the later generations of *CaRZFP1*-transgenic lettuce (Fig. 1g–i). Overall, growth of *CaRZFP1*-transgenic lettuce plants was dramatically impeded by *CaRZFP1* expression in a dose-dependent fashion.

This negative correlation extended to the root growth. Transgenic lettuce possessed shorter, less-developed roots, and this phenotype was closely correlated to *CaRZFP1* transcript levels. Compared with vector-only plants, primary root growth as well as total root-system length and lateral/adventitious root formation were strongly decreased in line no. 12 and mildly impaired in line no. 14, no. 15, and no. 16 but only slightly affected in line no. 6 (Fig. 1j). Average root fresh weights in line no. 6, no. 12, no. 14 no. 15, and no. 16 were 1.05 ± 0.15, 0.17 ± 0.01, 0.55 ± 0.09, 0.39 ± 0.04, and 0.52 ± 0.08 g, while those in V10, V30, and V38 were 1.71 ± 0.20, 1.64 ± 0.28, and 1.40 ± 0.28 g (Fig. 1k). Thus, *CaRZFP1* expression was strongly correlated with root underdevelopment (Fig. 1k and l). Again, *CaRZFP1* expression in line no. 6 was very low in the root and required confirmation with RT-PCR and DNA blot analysis (Fig. 1m, n).

**Fig. 2:**
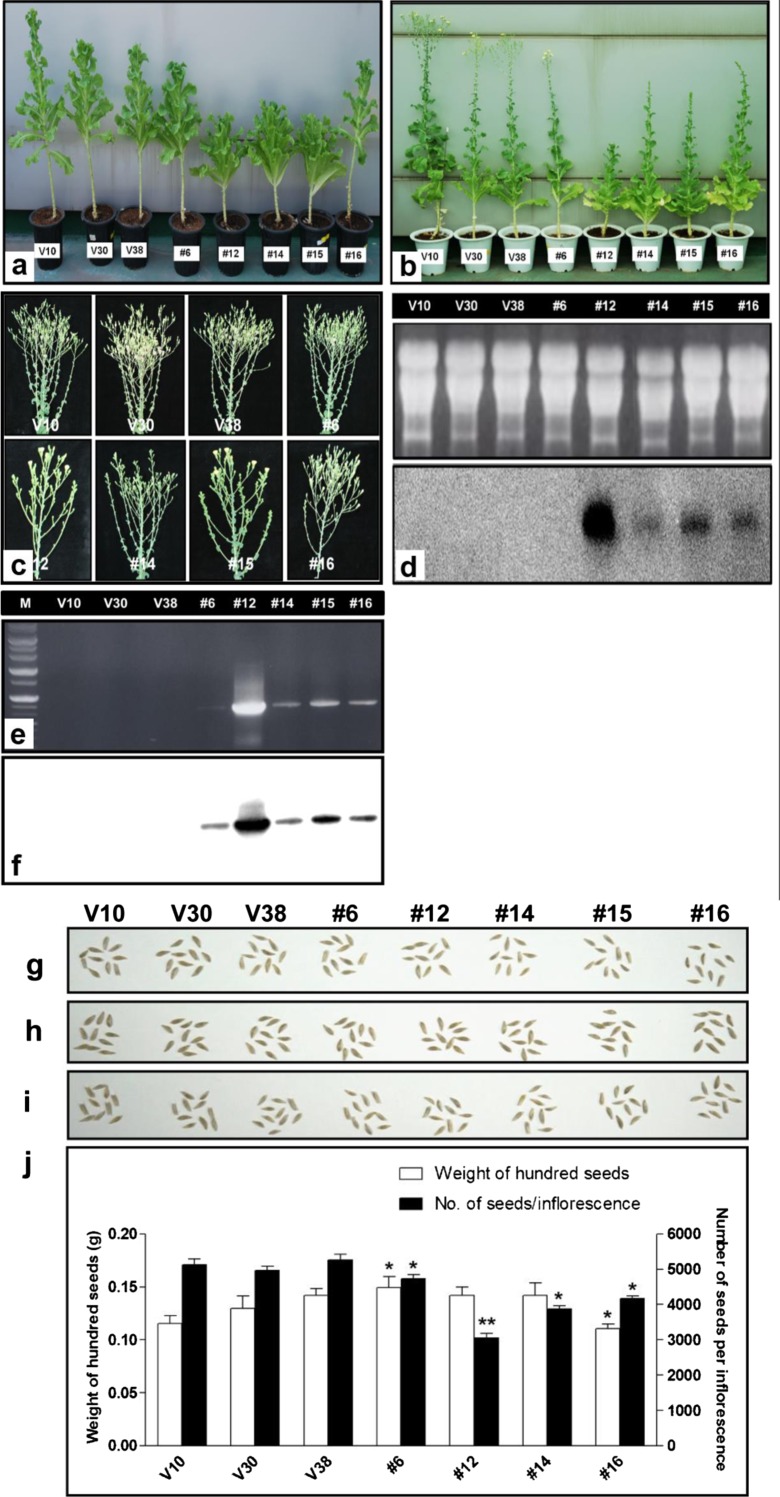
Hampered growth and development CaRZFP1 overexpressing transgenic lettuce plants extended to the reproductive stage. **a** Typical examples of T3 transgenic lettuce plant lines no. 6, no. 12, no. 14, no. 15 and no. 16 and lettuce plants carrying only the vector at the full growth. **b** Typical examples of T3 transgenic lettuce plant lines and vector-control lines at the flowering stage. **c** Fully developed inflorescences. **d** RNA blot hybridization results for the plants in **b**. **e** RT-PCR results for the same samples in **d**. **f** RT-PCR results in **e** was further confirmed by DNA blot analysis with ^32^P-labeled CaRZFP1 probe. M, size marker. **g** Typical examples of mature T2 generation seeds. **h** Typical examples of mature T3 generation seeds. **i** Typical examples of mature T4 generation seeds. **j** Average weight of 100 mature seeds and number of seeds per inflorescence of CaRZFP1-transgenic and vector-only lettuce lines from T2, T3 and T4 generation. Error bars show standard deviation. Student *t* test analyses showed that there was statistically significant at the <0.05 and < 0.001 level (*P < 0.05 and **P < 0.001) difference between the average weight of 100 mature seeds and number of seeds per inflorescence of CaRZFP1-transgenic and vector-only lettuce lines

The weak growth of *CaRZFP1*-transgenic lettuce plants continued late in development, in contrast to vector-only plants, which appeared normal from the vegetative phase to flowering. At full growth, *CaRZFP1*-transgenic lettuce was shorter than vector-only plants (Fig. 2a). Additionally, vector-only plants began to flower at 115 days after sowing (DAS), whereas *CaRZFP1*-transgenic lettuce began to flower 123 to 130 DAS (Fig. 2b). Inflorescence size was smaller in transgenic lettuce than in vector-only plants (Fig. 2c). Flower size did not differ significantly between the transgenic and control lines, but the former had significantly fewer flowers per inflorescence, a characteristic that was again correlated with *CaRZFP1* transcript levels. Transgenicity at full growth was again confirmed with RNA blot hybridization for most lines and with RT-PCR and DNA blot analysis for line no. 6 (Fig. 2d–f). Seed development in each flower appeared normal in transgenic lettuce (Fig. 2g–i), although the total seed number per inflorescence was negatively correlated with *CaRZFP1* expression levels. Seed size, morphology, and weight in the transgenic lettuce lines did not differ significantly from those in the vector-only plants (Fig. 2g–j). We next selected four *CaRZFP1*-transgenic lettuce lines (no. 6, no. 12, no. 14, and no. 16) for further investigation.

### *CaRZFP1*-transgenic lettuce plants were differentially damaged in root internal structures

**Fig. 3:**
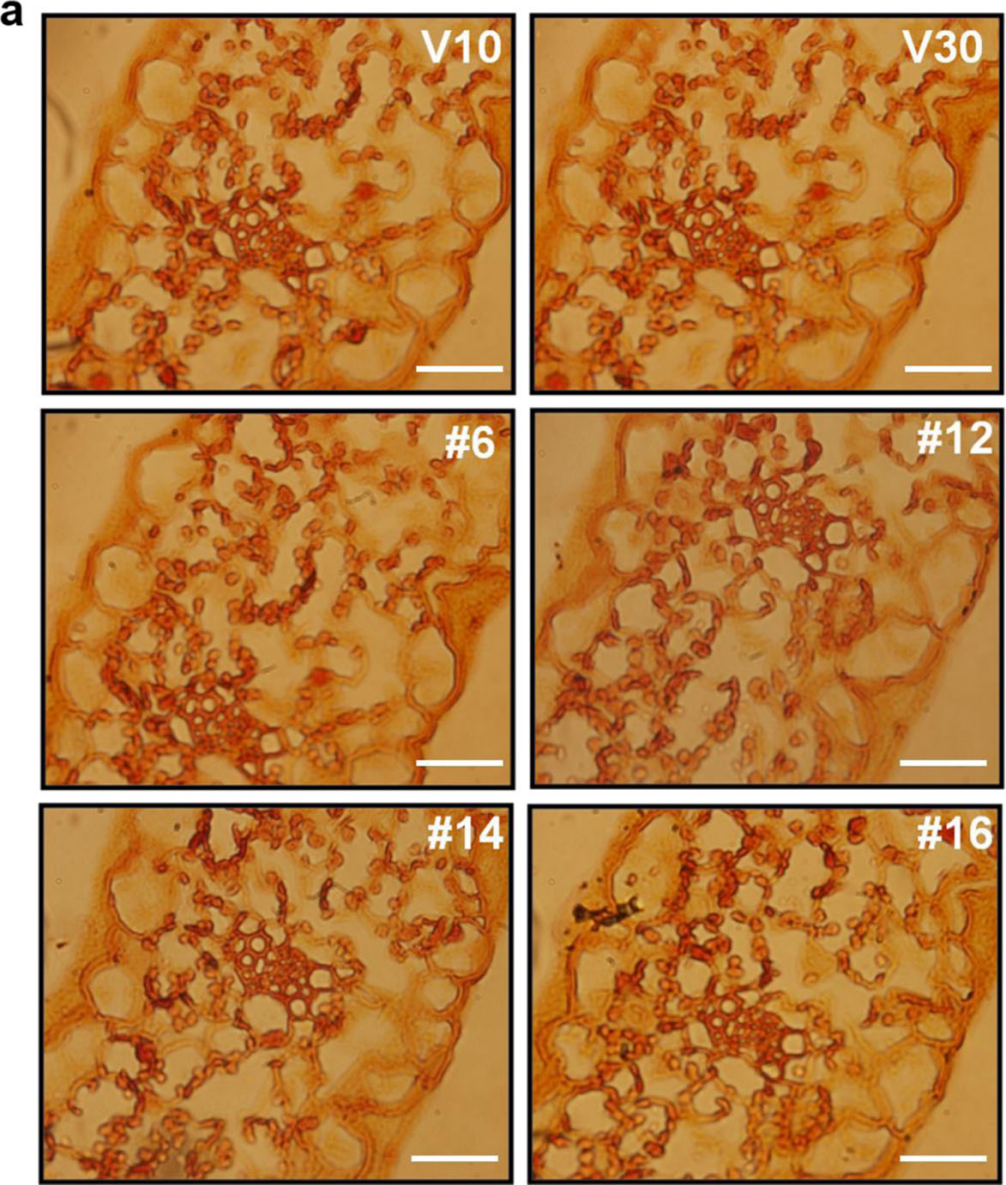

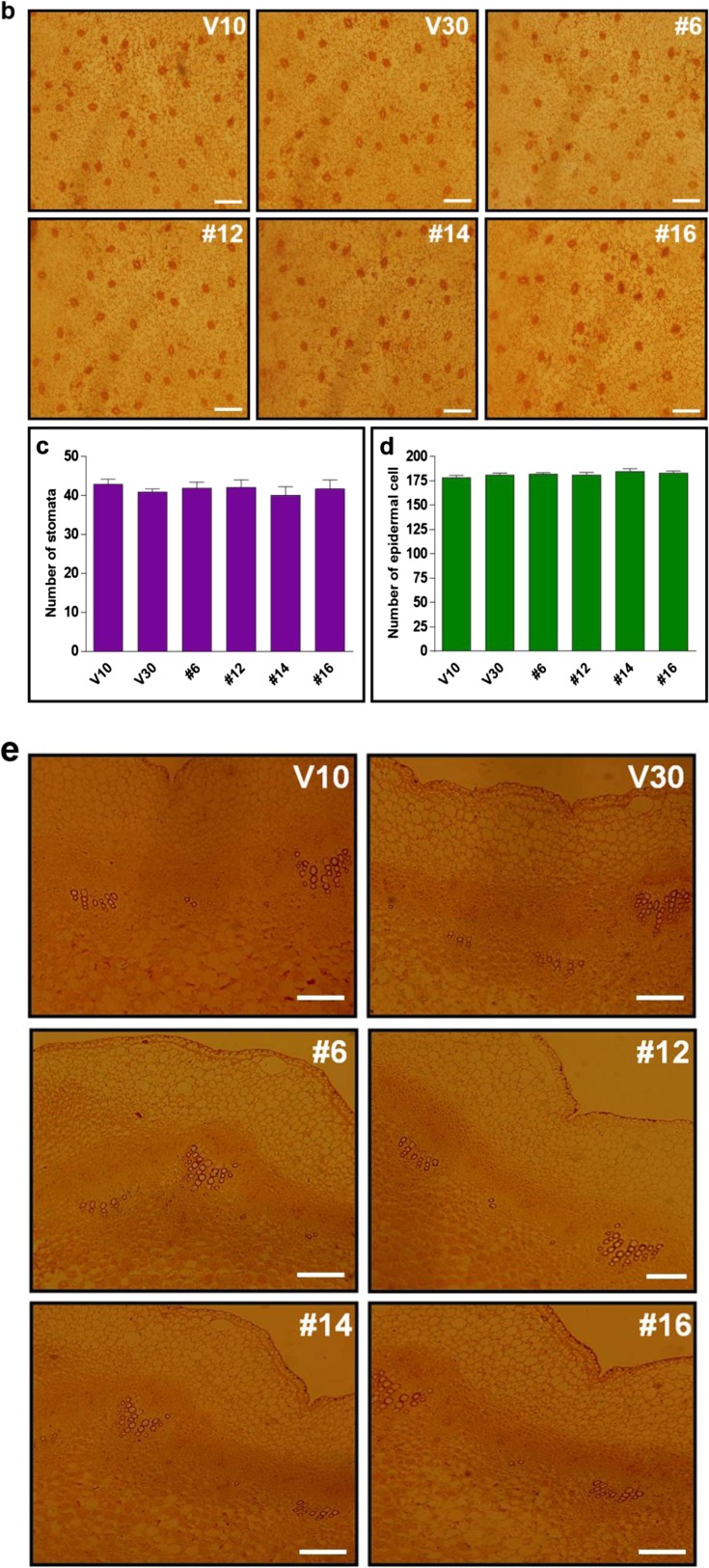

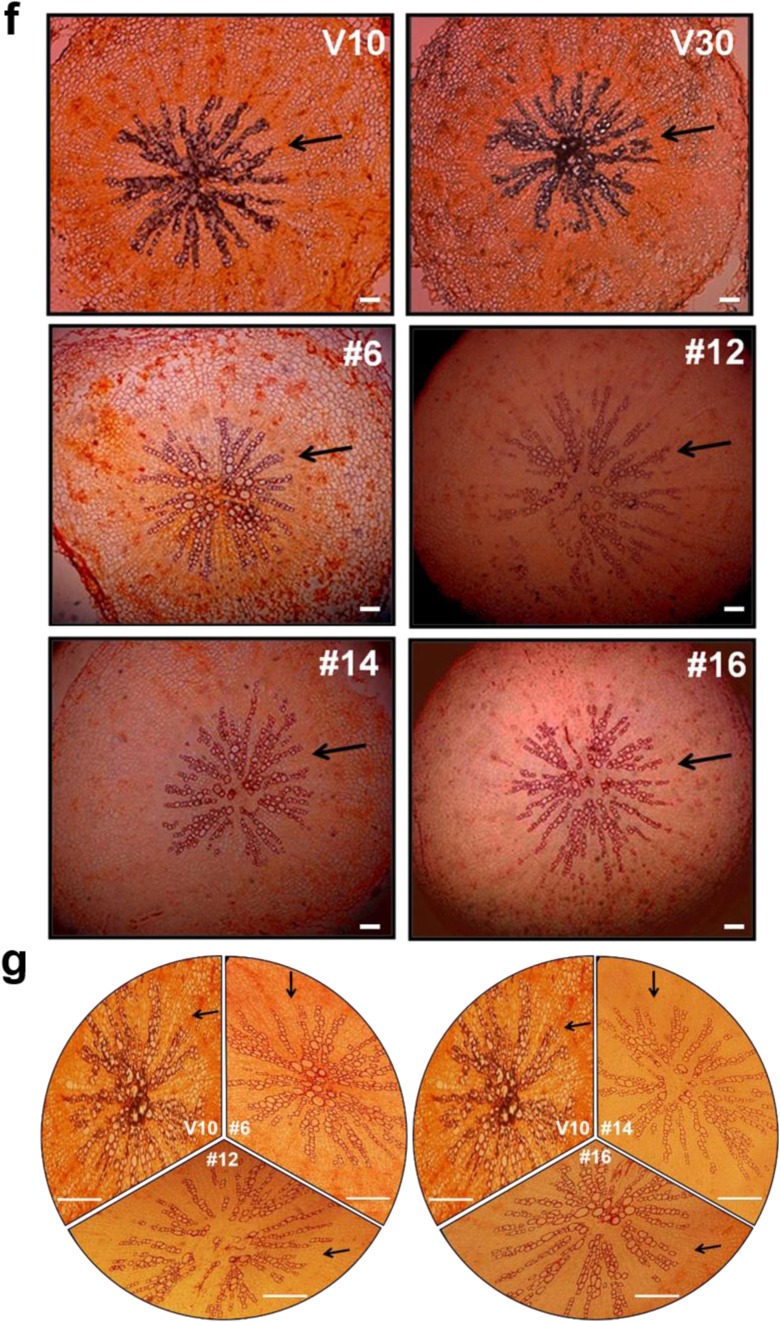

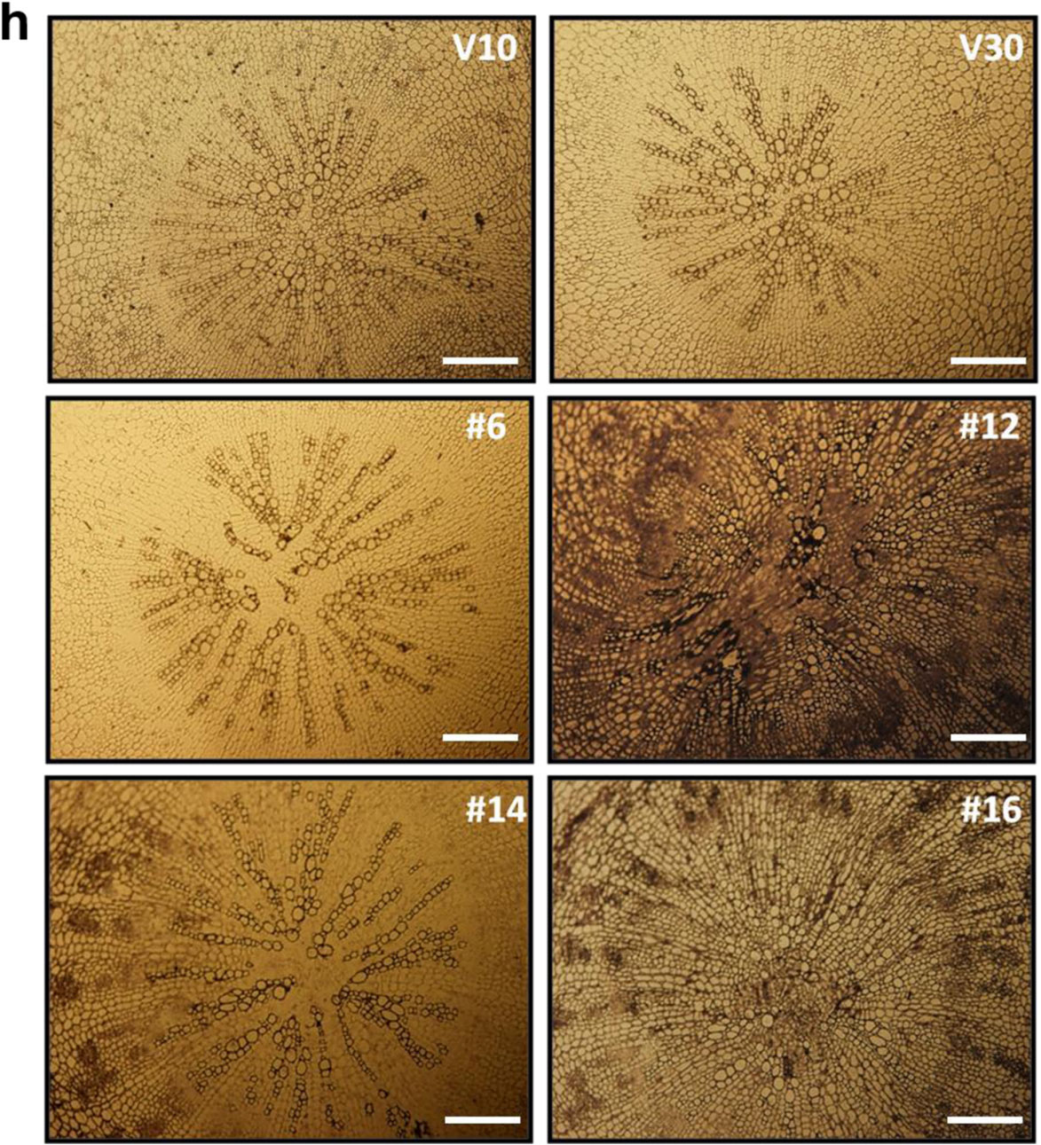
Cytological comparisons of *CaRZFP1*-transgenic and vector-only lettuce plants. **a** Leaf cross sections of *CaRZFP1*-transgenic lettuce and vector-only lettuce. Scale bar is 10 μm. **b** Leaf epidermal layer with stomata. Scale bar is 25 μm. **c** Number of stomata in *CaRZFP1*-transgenic lettuce lines and vector-only lettuce lines. **d** Number of epidermal cells in *CaRZFP1*-transgenic lettuce lines and vector-only lines. Error bars show standard deviation. **e** Stem cross sections of *CaRZFP1*-transgenic lettuce lines and vector-only lines. Scale bar is 25 μm. **f** Root cross sections of *CaRZFP1*-transgenic lettuce lines and vector-only lines. Arrows point endodermis layer. Scale bar is 25 μm. **g** Magnified view of **f**. Scale bar is 25 μm. **h** In situ localization of *CaRZFP1* transcript in *CaRZFP1*-transgenic lettuce plant roots. Cross sections of roots were hybridized with digoxigenin-labeled *CaRZFP1* antisense RNA probes. Scale bar is 25 μm

To examine the retarded growth of *CaRZFP1*-transgenic lettuce and the robust growth of *CaRZFP1*-transgenic tobacco (Zeba et al. 2009) on the cellular level, we analyzed leaf, stem, and root sections of both plants and compared them with vector-only controls. Leaves of transgenic lettuce and vector-only lettuce were not distinguishable in terms of morphology, cell size, or tissue organization. Cross sections of transgenic and control lines revealed basically identical mesophyll cell size and number, vascular bundle structure, chloroplast distribution in the mesophyll cells, as well as epidermal cell size and morphology (Fig. 3a). Top views of epidermal cells and stomata of transgenic and vector-only lettuce did not reveal any differences in epidermal cell size, stomatal size, or stomatal density (Fig. 3b–d). Likewise, stem morphology, cell size, and tissue organization in transgenic versus vector-only lettuce were indistinguishable (Fig. 3e). Significant defects in endodermis and vascular bundle development were observed in *CaRZFP1*-transgenic lettuce roots. The internal structure of vector-only lettuce roots was typical and well defined; visible structures included the surrounding epidermal layer, parenchyma cells inside the epidermis, endodermal layer, and pericycle surrounding the internal vascular bundles, as well as radially lined vessel elements and phloem between the xylem elements (Fig. 3f, g). The internal root structures of transgenic lettuce plants significantly deviated from typical. In line with the strongest *CaRZFP1* expression (no. 12), the endodermis and pericycle were barely defined and vessel elements were not compactly structured and sometimes even disconnected, while the xylem element did not appear to be formed at the root center (Fig. 3f, g). These root development defects were also present in other transgenic lettuce lines to a lesser extent and were strongly correlated with *CaRZFP1* expression levels; stronger *CaRZFP1* expression resulted in greater impairment in endodermis, pericycle, and vascular bundle structure. However, *CaRZFP1* expression did not seem to affect root cortex cells; parenchymal cell size and overall shape were normal in *CaRZFP1*-transgenic lettuce (Fig. 3f, g). To identify spatial *CaRZFP1* expression patterns in *CaRZFP1*-transgenic and vector-only lettuce roots, we performed in situ hybridization assays. The antisense probe of *CaRZFP1* detected the transcript strongly from the roots of line no. 12, at medium levels from line no. 14 and no. 16 and at low levels from line no. 6; however, *CaRZFP1* mRNA was expressed equally within all root cell types in every transgenic plant. In the vector-only plants, no significant signal could be detected (Fig. 3h). Hybridization of the root sections with the *CaRZFP1* sense probe found no significant signal over background, failing to distinguish between the transgenic and vector-only roots (data not shown). Previously, we engineered transgenic lines of tobacco overexpressing *CaRZFP1* under the control of CaMV35S promoter. These *CaRZFP1*-transgenic tobacco plants exhibited robust growth and abiotic stress tolerance (Zeba et al. 2009). When these *CaRZFP1*-transgenic tobacco plants were examined for the development of roots at the cellular level, the overall root cell structure, tissue patterns, or morphology, including the endodermis and vascular bundle, did not differ from vector-only plants (Fig. S2a–c).

### Different genes were induced in *CaRZFP1*-transgenic lettuce and *CaRZFP1*-transgenic tobacco plants

To elaborate on the potential underlying genes, whole transcriptome profiling was performed on transgenic lettuce that differed in *CaRZFP1* transcript levels (low, moderate, and high; line no. 6, no. 12, no. 14, and no. 16) and the vector-only lettuce. Agilent Arabidopsis GE 4 X 44K microarray was used for the transcriptome profiling of the lettuce lines because a lettuce microarray was unavailable and our previous work on tobacco also used the Arabidopsis microarray (Zeba et al. 2009). Differentially expressed (either up- or down-regulated) transcripts between line no. 12 (with the strongest *CaRZFP1* expression) and control lines were determined via a twofold change threshold (*P* < 0.05). Among 87 genes identified as significantly differentially expressed, 73 were up-regulated (Tables 1a and S1) and 14 were down-regulated (Tables 1b and S2). Differentially expressed genes in transgenic line no. 12 were again screened for the correlation between *CaRZFP1* expression and growth impairment in line no. 14, no. 16 (medium *CaRZFP1* expression), and no. 6 (low *CaRZFP1* expression). This analysis verified the presence of correlative change in fold values from the highest in line no. 12 to the lowest in vector-only lines. We separated the up- and down-regulated genes into two groups: “Group 1” included genes with correlative changes in expression level among all four *CaRZFP1*-transgenic lines, while “Group 2” included only genes with significant expression level changes mainly in line no. 12. Group 1 contained 24 up-regulated putatively annotated genes, seven up-regulated unannotated genes, seven down-regulated putatively annotated genes, and one down-regulated unannotated gene (Tables 1a, b, and S1 and S2). Group 2 contained 39 up-regulated putatively annotated genes, three up-regulated unannotated genes, four down-regulated putatively annotated genes, and two down-regulated unannotated genes (Tables 1a, b, and S1 and S2).

**Table 1:** Transcriptome profiling of *CaRZFP1* overexpressing lettuce and tobacco plants by microarray analysis

**Gene symbol**	**Gene description**	***CaRZFP1*** **-transgenic lettuce lines/vector controls (log**_**2**_ **fold change)**			
		**No. 6**	**No. 14**	**No. 16**	**No. 12**
a. Genes up-regulated in *CaRZFP1*-overexpressing T_4_ generation lettuce plants					
**At5g53170**	**Cell division protease ftsH-11 (FTSH11)**	**0.28**	**0.38**	**1.36**	**2.36**
**At5g52910**	**Timeless family protein (ATIM)**	**0**	**0.52**	**0.42**	**3.35**
At1g08260	DNA polymerase epsilon subunit 1 (TIL1)	0	0	0	3.35
At1g80490	Topless-related protein 1 (TPR1)	0.50	0.75	0.37	3.31
**At1g55110**	**Indeterminate-domain 7 protein (IDD7)**	**0.03**	**1.33**	**0.76**	**3.09**
**At4g31920**	**Arabidopsis response regulator 10 (ARR10)**	**0.00**	**0.30**	**0.11**	**3.31**
**At5g08130**	**BES1-interacting MYC-like 1 (BIM1)**	**0**	**0.20**	**0.18**	**3.08**
At1g80580	Ethylene-responsive factor (ERF)	0	0	0	4.32
At1g08465	Putative axial regulator YABBY 2 (YAB2)	0	0	0	5.97
At1g20910	ARID/BRIGHT DNA-binding domain-containing protein	0	0	0	4.38
**At3g44370**	**Membrane insertion protein, OxaA/YidC with tetratricopeptide repeat domain-containing protein**	**0.21**	**1.23**	**1.01**	**2.68**
**At1g21310**	**Extensin 3 (EXT3)**	**0.73**	**1.44**	**1.02**	**3.50**
At4g33610	Glycine-rich protein	0	0	0	3.08
At3g28550	Proline-rich extensin-like family protein	0	0	0	3.04
At3g24860	Hydroxyproline-rich glycoprotein family protein	0	0	0	2.95
**At4g30170**	**Peroxidase 45**	**0.12**	**1.34**	**0.83**	**2.65**
**At5g48300**	**Glucose-1-phosphate adenylyltransferase small subunit (ADG1)**	**0.02**	**0.26**	**0.41**	**2.79**
**At5g08530**	**NADH dehydrogenase (ubiquinone) flavoprotein 1(CI51)**	**0.02**	**1.22**	**1.53**	**3.02**
**At3g03890**	**FMN binding protein**	**1.39**	**1.59**	**2.08**	**3.12**
At4g10120	Sucrose-phosphate synthase	0	0	0	2.44
At5g66230	Chalcone-flavanone isomerase family protein	0	0	0	4.34
At1g17420	Lipoxygenase 3 (LOX3)	0	0	0	4.60
At5g08100	Isoaspartyl peptidase/l-asparaginase 1 subunit beta	0	0	0	4.63
At5g41080	Glycerophosphoryl diester phosphodiesterase family protein	0	0	0	4.10
At1g54620	Pectin methylesterase inhibitor superfamily protein	0	0	0	4.30
At1g06700	Protein kinase domain-containing protein	0.50	0.48	0.06	4.08
**At3g48190**	**Serine/threonine-protein kinase (ATM)**	**0.00**	**1.50**	**0.86**	**2.74**
At4g04960	L-type lectin receptor kinase VII.1 (LECRK-VII.1)	0	0	0	4.31
At3g20860	NIMA-related kinase 5 (NEK5)	0	0	0	2.44
At3g24540	Proline-rich extensin-like receptor kinase (PERK)	0	0	0	3.46
At5g11360	Interleukin-1 receptor-associated kinase 4 protein	0	0	0	3.33
At5g12235	CLAVATA3/ESR-related 22 protein (CLE22)	0	0	0	3.99
**At3g55840**	**Hs1pro-1 protein**	**0.54**	**1.16**	**0.87**	**4.62**
At3g61185	Defensin-like (DEFL) family protein	0	0	0	5.43
**At4g10850**	**Nodulin MtN3-like protein**	**0.32**	**0.40**	**0.39**	**3.39**
**At3g07100**	**Sec24-like transport protein (ERMO2)**	**0.69**	**1.27**	**2.14**	**3.49**
**At5g27100**	**Glutamate receptor 2.1 (GLR2.1)**	**0.06**	**0.24**	**1.97**	**2.52**
**At5g01990**	**Auxin efflux carrier family protein**	**0.87**	**1.44**	**1.48**	**2.32**
At3g26520	Tonoplast intrinsic protein 2 (TIP2)	0	0	0	2.86
At2g39890	Proline transporter 1 (PROT1)	0	0	0	3.27
At5g51710	K(+) efflux antiporter 5 (KEA5)	0	0	0	2.47
At1g79520	Cation efflux family protein	0	0	0	2.47
At3g55740	Proline transporter 2 (PROT2)	0	0	0	2.55
At1g80300	Nucleotide transporter 1 (NTT1)	0	0	0	3.64
At1g23910	Polyketide cyclase/dehydrase and lipid transport superfamily protein	0	0	0	4.77
At4g19680	Fe(2+) transport protein 2 (IRT2)	0	0	0	4.06
At5g59030	Copper transporter 1 (COPT1)	0	0	0	4.65
At4g08290	Nodulin MtN21/EamA-like transporter family protein	0	0	0	4.88
**At1g31920**	**Pentatricopeptide repeat-containing protein**	**0.14**	**1.39**	**1.29**	**3.76**
**At2g17600**	**Cysteine/histidine-rich C1 domain-containing protein**	**0.46**	**0.62**	**0.48**	**2.77**
**At5g45428**	**Conserved peptide upstream open reading frame 24 (CPuORF24)**	**0.53**	**0.54**	**0.63**	**3.23**
**At5g42990**	**Putative ubiquitin-conjugating enzyme E2 18 (UBC18)**	**0.27**	**0.77**	**1.01**	**2.17**
**At1g71410**	**Armadillo/beta-catenin-like repeats-containing protein**	**0.75**	**1.88**	**2.04**	**3.09**
**At4g01640**	**F-box associated ubiquitination effector family protein**	**0.04**	**0.66**	**0.80**	**6.21**
**At2g35140**	**Development and cell death domain protein (DCD)**	**0**	**0.31**	**0.88**	**3.53**
At1g31090	F-box domain-containing protein	0	0	1.26	2.97
At4g03510	E3 ubiquitin-protein ligase (RMA1)	0	0	2.05	3.47
At3g62940	Cysteine proteinases family protein	0	0	0.17	4.72
At3g26805	Aspartic protease family protein	0	0	0	4.47
At4g30020	PA-domain-containing subtilase family protein	0	0	0	4.99
At1g06630	F-box domain-containing protein	0	0	0	3.43
At3g23880	F-box/kelch-repeat protein	0	0	0	3.69
At3g11000	Development and cell death domain protein (DCD)	0	0	0	5.13
**At2g18970**	**Uncharacterized gene**	**0.83**	**1.92**	**0.95**	**3.40**
**At5g04550**	**Uncharacterized gene**	**0.11**	**1.43**	**1.67**	**3.29**
**At1g55160**	**Uncharacterized gene**	**0.63**	**1.13**	**1.86**	**3.54**
**At2g11630**	**Uncharacterized gene**	**0.02**	**0.23**	**0.59**	**6.04**
**At2g24755**	**Uncharacterized gene**	**0**	**0.32**	**0.51**	**4.97**
**At5g26775**	**Uncharacterized gene**	**0**	**1.02**	**1.71**	**6.71**
**At3g11300**	**Uncharacterized gene**	**0**	**0**	**1.09**	**4.25**
At1g43195	Uncharacterized gene	0	0	0	4.21
At2g22122	Uncharacterized gene	0	0	0	4.47
At1g63610	Uncharacterized gene	0	0	0	4.38
b. Genes down-regulated in *CaRZFP1*-overexpressing T_4_ generation lettuce plants					
At2g47850	Zinc finger CCCH domain-containing protein 32	− 0.90	− 0.54	− 0.43	− 3.62
**At4g10300**	**RmlC-like cupins super family protein**	**− 0.85**	**− 0.92**	**− 1.69**	**− 2.02**
**At3g55850**	**Amidohydrolase family protein (LAF3)**	**− 0.12**	**− 0.19**	**− 0.51**	**− 2.63**
**At4g15550**	**Indole-3-acetate beta-D-glucosyltransferase (IAGLU)**	**− 0.05**	**− 0.15**	**− 0.11**	**− 3.41**
At5g37600	Glutamine synthetase cytosolic isozyme 1–1 (GSR 1)	− 1.68	− 1.31	− 1.85	− 3.22
**At3g44200**	**Serine/threonine-protein kinase Nek5 (NEK6)**	**− 1.44**	**− 1.46**	**− 1.92**	**− 2.06**
**At1g60800**	**NSP-interacting kinase 3 (NIK3)**	**−**− **1.30**	**−**− **1.32**	− **1.63**	− **2.96**
**At2g47990**	**Transducin family protein/WD-40 repeat family protein**	**− 0.59**	**− 0.92**	**− 1.65**	**− 2.00**
At4g08950	Phosphate-responsive 1 family protein (EXO)	− 1.29	− 1.35	− 1.09	− 4.47
At3g02490	Pentatricopeptide repeat (PPR) superfamily protein	− 1.40	− 0.75	− 0.81	− 3.11
**AtCG00290**	**tRNA-Ser**	**− 0.17**	**− 0.74**	**− .074**	**− 2.64**
**At5g37650**	**Uncharacterized gene**	**− 0.71**	**− 1.07**	**− 1.38**	**− 3.17**
At3g52742	Uncharacterized gene	− 0.41	− 1.10	− 0.12	− 2.14
At3g15518	Uncharacterized gene	− 2.26	− 2.12	− 1.73	− 3.95
**Gene symbols**	**Gene description**	***CaRZFP1*** **-transgenic tobacco/vector controls (log**_**2**_ **fold change)**			
c. Up-regulated genes in *CaRZFP1-*overexpressing T_2_ generation tobacco plants (Zeba et al. 2009)					
At5g55280	Cell division protein ftsZ	2.21			
At3g12400	Tumor susceptibility gene 101 (tsg101) family protein	3.21			
At1g70490	ADP-ribosylation factor	6.88			
At1g13740	ABI five binding protein 2	5.80			
At5g39650	DUO1-activated unknown 2 (DUO2)	2.03			
At5g10430	Arabinogalactan-protein (agp4)	4.42			
At5g64310	Arabinogalactan-protein (agp1)	2.52			
At4g37450	Arabinogalactan-protein (agp18)	3.50			
At2g46330	Arabinogalactan-protein (agp16)	2.87			
At5g49080	Proline-rich extensin-like family protein	2.18			
At3g49300	Proline-rich family protein	1.78			
At3g49305	Hypothetical protein contains proline-rich extensin domains	2.17			
At4g38770	Proline-rich family protein (prp4)	6.05			
At4g08380	Proline-rich extensin-like family protein	2.48			
At5g19800	Hydroxyproline-rich glycoprotein family protein	2.72			
At2g05380	Glycine-rich protein (grp3)	3.71			
At1g11580	Pectin methylesterase	2.32			
At5g38410	Ribulose bisphosphate carboxylase small chain 3b	3.49			
At3g23820	NAD-dependent epimerase	2.21			
At3g25140	Glycosyl transferase family 8 protein	2.86			
At4g15233	ABC transporter family protein	7.31			
At1g11860	Aminomethyltransferase	2.72			
At5g58050	Glycerophosphoryl diester phosphodiesterase family protein	2.43			
At4g13090	Xyloglucan:xyloglucosyl transferase/xyloglucan endotransglycosylase	2.27			
At3g63200	Patatin-like protein 9	4.88			
At1g80380	Phosphoribulokinase/uridine kinase-related	2.51			
At4g36360	Beta-galactosidase	4.55			
At2g46820	Curvature thylakoid 1B (CURT1B)	3.07			
At1g74670	Gibberellin-responsive protein	4.46			
At2g45770	Signal recognition particle receptor protein/chloroplast (ftsY) similar to cell division protein	3.06			
At1g55480	Plant protein family containing a PDZ, a K-box, and a TPR motif (ZKT)	2.61			
At5g48760	60s ribosomal protein L13A (RPL13aD)	1.89			
At3g59760	Cysteine synthase c/O-acetylserine (thiol)-lyase isoform c	2.22			
At2g17360	40s ribosomal protein s4 (rps4a)	2.32			
At1g02780	60s ribosomal protein	3.31			
At5g13650	Elongation factor family protein	2.38			
At4g33510	3-Deoxy-D-arabino-heptulosonate-7-phosphate 2 (dahp2)	2.58			
At5g64680	Uncharacterized gene	2.23			
At5g36070	Uncharacterized gene	5.56			
At3g43684	Uncharacterized gene	2.58			
At2g36885	Uncharacterized gene	4.09			
At2g05752	Uncharacterized gene	4.38			
At5g02240	Uncharacterized gene	3.00			
At1g67700	Uncharacterized gene	1.99			
At4g18070	Uncharacterized gene	2.28			
d. Down-regulated genes in *CaRZFP1-*overexpressing T_2_ generation tobacco plants (Zeba et al. 2009)					
At1g61780	Postsynaptic protein-related	− 1.94			
At5g60870	Regulator of chromosome condensation (rcc1) family protein	− 2.43			
At3g12090	Tetraspanin gene family (TET6)	− 2.43			
At5g66070	Zinc finger (C3HC4-type ring finger) family protein	− 2.03			
At1g54060	Trihelix DNA-binding protein family	− 1.80			
At4g02720	NF-kB activating protein (NKAP) proteins	− 2.22			
At5g02820	Brassinosteroid insensitive 5 (BIN5)	− 2.55			
At3g13520	Arabinogalactan-protein (agp12)	− 2.36			
At2g05540	Glycine-rich protein	− 1.69			
At1g29050	Trichome birefringence-like 38	− 2.25			
At2g18700	Trehalose phosphatase/synthase 11 (TPS11)	− 2.40			
At2g18600	Rub1-conjugating enzyme	− 2.23			
At3g47950	ATPase	− 2.03			
At3g19700	Leucine-rich repeat transmembrane protein kinase	− 2.17			
At5g63870	Serine/threonine-protein phosphatase (pp7)	− 2.16			
At5g56460	Protein kinase	− 2.86			
At5g60460	Sec61-beta subunit family protein	− 2.78			
At2g37470	Histone h2b	− 3.19			
At4g27960	Ubiquitin-conjugating enzyme 9 (ubc9)	− 2.35			
At3g01400	Armadillo/beta-catenin repeat family protein	− 2.89			
At5g66040	Protein with thiosulfate sulfurtransferase/rhodanese activity (STR16)	− 1.91			
At3g09440	Heat shock protein 70 (hsp70)	− 1.83			
At2g46550	Uncharacterized gene	− 2.66			
At1g63930	Uncharacterized gene	− 2.72			

Group 1 genes are in bold

To validate the transcriptome analysis results, total RNA from the same transgenic lettuce lines used for transcriptome experiments was subjected to RNA blot analyses with oligonucleotides corresponding to those on the microarray. Nine genes that were either significantly up- or down-regulated in transgenic lettuce lines were randomly selected for analysis. The genes encoded an FMN binding protein, Hs1pro-1 protein, *EXT3*, *F-box*, auxin efflux carrier protein, *ERMO2*, *ATM*, *NIK3*, and *NEK6*. Although some variation existed between the microarray and RNA blot results, trends in the differentially expressed genes were generally consistent across the two different approaches (Fig. S3).

## Discussion

Genes significantly up-regulated in *CaRZFP1-*transgenic lettuce compared with vector-only plants could be grouped into two, Group 1 and Group 2. Among Group 1 genes, cell division protease ftsH-11 (FTSH11) has been implicated in housekeeping proteolysis of membrane proteins (Wagner et al. 2016). Timeless family protein (ATIM) regulates circadian rhythm mechanism in the files (Rosato et al. 2006). Indeterminate-domain (IDD) protein family members have been implicated in auxin production, gravitropism and lateral organ differentiation, heat stress responses, regulation of sugar transporter regulation, as well as promotion of seed germination (Cui et al. 2013). In maize and rice, the *ID1* gene, a member of IDD family, acts as a master switch for transitioning from the vegetative to the reproductive phase. Plants with loss-of-function *id1* remain in a prolonged state of vegetative growth and form aberrant flowers (Park et al. 2008). Flowering time and inflorescence development were significantly altered in *CaRZFP1*-transgenic lettuce. Arabidopsis response regulator 10 (ARR10) has been implicated in the cytokinin signaling (To et al. 2007). BES1-interacting MYC-like 1 (BIM1) played an essential role in brassinosteroid signaling (Xing et al. 2013). The ectopic expression of *CaRZFP1* may regulate phytohormone signaling and subsequently affect the growth and development of *CaRZFP1-*transgenic lettuce plants. OxaA/YidC is essential for protein insertion into bacterial and mitochondrial inner membranes, as well as thylakoid membranes of chloroplasts (Hennon et al. 2015). Extensin 3 (EXT3) expression was also up-regulated over threefold in *CaRZFP1*-transgenic lettuce; these proteins are critical structural components of the cell wall during plant growth and development (Lamport et al. 2011). Peroxidases are among the largest protein families; they function in the cross-linking of cell wall proteins, lignin biosynthesis, suberization, auxin catabolism, oxidative stress, and defense responses (Marjamaa et al. 2009). Glucose-1-phosphate adenylyltransferase small subunit (ADG1) is a key regulatory enzyme in the plant starch and bacterial glycogen biosynthesis pathway (Bahaji et al. 2011). NADH:ubiquinone oxidoreductase in the Complex I of the electron transport chain is a central component in cellular respiration, the main process providing energy in most heterotrophic eukaryotes and in autotrophic organisms during their heterotrophic phase (Kuhn et al. 2015). FMN binding proteins are critical in the electron transport process. Overexpression of FMN binding protein (AtHal3) altered growth rates while improving salt and drought tolerance in Arabidopsis (Espinosa-Ruiz et al. 1999). The ectopic expression of *CaRZFP1* may deregulate genes involved in vital metabolic processes. Transcriptome analysis also showed that many genes with known function in plant development were altered in the *CaRZFP1-*transgenic lettuce. A serine/threonine-protein kinase (ATAXIA-TELANGIECTASIA MUTATED, ATM) that is important in DNA damage response (Liu et al. 2015) was also elevated in *CaRZFP1-*transgenic lettuce plants. The up-regulated Hs1pro-1 gene has been implicated in defense response in plants (Yuan et al. 2008). Nodulin MtN3-like protein is member of Sugars Will Eventually be Exported Transporters (SWEETs) family that is essential for the maintenance of animal blood glucose levels, plant nectar production, plant seed, and pollen development (Chandran 2015). Another significantly up-regulated gene in transgenic lettuce encoded sec24-like transport protein (ERMO2); *SEC24A* encodes a coat protein complex II vesicle coat subunit involved in endoplasmic reticulum-to-Golgi trafficking during the early secretory pathway. In plants, secretory pathway defects often lead to cell division defects (Qu et al. 2014). Glutamate receptor 2.1 (GLR2.1) is a member of ligand-gated ion channel family, and it functions in coordination of mitotic activity during root development, sensing carbon-to-nitrogen status, cellular calcium ion homeostasis, response to light, regulation of plant hormone biosynthesis, and signaling pathways (Weiland et al. 2016). Because an optimum auxin concentration is required for shoot and root growth, lateral root development, and differentiation of vascular strands (Aremu et al. 2016), excessive expression of auxin efflux carrier family genes could be harmful for plant growth and development. Pentatricopeptide repeat proteins function in multiple processes as modular RNA-binding proteins that mediate gene expression through altering RNA sequence, turnover, and processing. They exhibit profound effects on organelle biogenesis and function; consequently, they are influential in major plant processes, including photosynthesis, respiration, development, and environmental responses (Manna 2015). Thus, the ectopic expression of *CaRZFP1* may trigger secondary effects that are critical to plant growth and development. Cysteine/histidine-rich C1 domain-containing protein also has been implicated for its important role in regulating plant growth and development (Hwang et al. 2014). Conserved peptide upstream open reading frame 24 (CPuORF24) regulates polyamine and sucrose concentrations in response to starvation (Hayden and Jorgensen 2007). Ubiquitin-conjugating enzyme E2 18 (UBC18), armadillo/beta-catenin-like repeats-containing protein (ARM), F-box associated ubiquitination effector family protein, and development and cell death domain protein (DCD), all have been implicated in ubiquitin-mediated protein degradation. Ubiquitin-mediated protein degradation plays a key regulatory role during plant growth and development, as well as being implicated in plant hormone signaling (Mudgil et al. 2004). Thus, CaRZFP1 might also function at the post-translational level in various developmental processes through ubiquitin-dependent protein degradation.

Some genes were significantly down-regulated (< 2-fold) in *CaRZFP1-*transgenic lettuce compared with vector-only plants. RmlC-like cupin super family proteins have both enzymatic and non-enzymatic functions, the former including decarboxylases, isomerases, epimerases, oxidoreductases, disomerases, dioxygenases, and hydrolases, the latter including auxin binding, seed storage, and nuclear transcription factors (Uberto and Moomaw 2013). Amidohydrolase family protein (LAF3) is involved in phytochrome A signal transduction (Hare et al. 2003). Indole-3-acetate beta-D-glucosyltransferase (IAGLU) plays a crucial role in auxin conjugation pathway and auxin metabolism (Jackson et al. 2002). The NIMA-related Kinase 6 (NEK6) in Arabidopsis organizes microtubules, thus regulating cellular expansion, directional growth of roots and hypocotyls, petiole elongation, cell file formation, and morphogenesis (Takatani et al. 2015). NSP-interacting kinase 3 (NIK3) is involved in plant defense response and developmental processes (Zorzatto et al. 2015). WD40 domains are present in eukaryotic proteins linked to scaffolding, cooperative assembly, chaperoning other proteins, and regulation of multicellular processes. WD40 repeat protein NEDD1 regulates microtubule development during mitotic cell division in Arabidopsis (Gachomo et al. 2014). The transcript level of tRNA-Ser was also significantly repressed in *CaRZFP1*-transgenic lettuce plants.

These 24 up-regulated genes and seven down-regulated Group 1 genes are all necessary for plant growth and development. Thus, the negative phenotypic results related to *CaRZFP1* expression in lettuce are probably caused by imbalance among growth and development regulators. Ectopically expressed CaRZFP1 induced and suppressed various genes in lettuce, leading to composite negative effects on growth and development, especially in the root and floral meristem division in the inflorescence. Although root endodermal development was significantly hampered in the CaRZFP1-transgenic lettuce plants, none of the annotated genes exhibited a direct relationship in their regulation with suberin biosynthetic processes, except one peroxidase that was expressed, albeit not significantly. We note that among Group 1, seven uncharacterized genes were correlatively up-regulated and one, correlatively down-regulated; it is impossible to speculate on their functions at present (Tables 1a, b, S1 and S2). In Group 2, 43 genes were strongly overexpressed and six genes strongly down-regulated in the line no. 12 (Tables 1a, b, S1 and S2). Because the regulation of these genes was not correlated with *CaRZFP1* expression in the line no. 14, no. 16, and no. 6, their overexpression and suppression in the line no. 12 were likely the result of composite effects from the biased expression of Group 1 genes, rather than due directly to *CaRZFP1* expression.

Ectopic expression of *CaRZFP1* in tobacco enhanced plant growth (larger leaves, longer hypocotyls, longer primary roots, and increased lateral roots), leading to heavier fresh weight (Zeba et al. 2009). Transcriptome analysis revealed that growth-related genes were widely altered, i.e., 37 up-regulated and 22 down-regulated annotated genes, in *CaRZFP1*-overexpressing transgenic tobacco (Tables 1c, d, S3 and S4). Up-regulated ADP-ribosylation factors are important in regulating intracellular membrane trafficking, a process linked to root development and the polar localization of PIN-FORMED (PIN) family auxin efflux facilitators (Yuan et al. 2015). Dramatic elevation (up to sixfold) was observed in the expression of growth-related cell wall proteins, four of which were arabinogalactan proteins (AGPs) (Showalter and Basu 2016). These results suggest that CaRZFP1 activates and positively regulates cell wall protein expression to modify cell wall plasticity, effectively promoting the growth of transgenic tobacco plants. Proline-rich proteins are important in Arabidopsis root-hair formation (Boron et al. 2014), and transcriptome analysis revealed that four proline-rich protein genes were up-regulated in *CaRZFP1*-transgenic tobacco, and *CaRZFP1*-transgenic tobacco plants exhibited more developed root hairs (Zeba et al. 2009). Plant ABC transporter family proteins experienced an over sevenfold up-regulation; these proteins are implicated in chlorophyll biosynthesis, Fe-S cluster formation, stomatal movement, and ion fluxes (Hwang et al. 2016). Gibberellin-responsive proteins were also highly induced in transgenic tobacco; these proteins have been implicated in hypocotyl and stem elongation (Achard et al. 2007). Ectopic expression of *CaRZFP1* in tobacco also down-regulated two dozens of genes but to a lesser degree than the up-regulation experienced by the genes described above (Tables 1d, and S4). Finally, eight up- and two down-regulated genes were unannotated in *CaRZFP1*-transgenic tobacco (Tables 1c, d, S3 and S4), and their possible effects on tobacco growth and development are not feasible to be suggested.

**Fig. 4:**
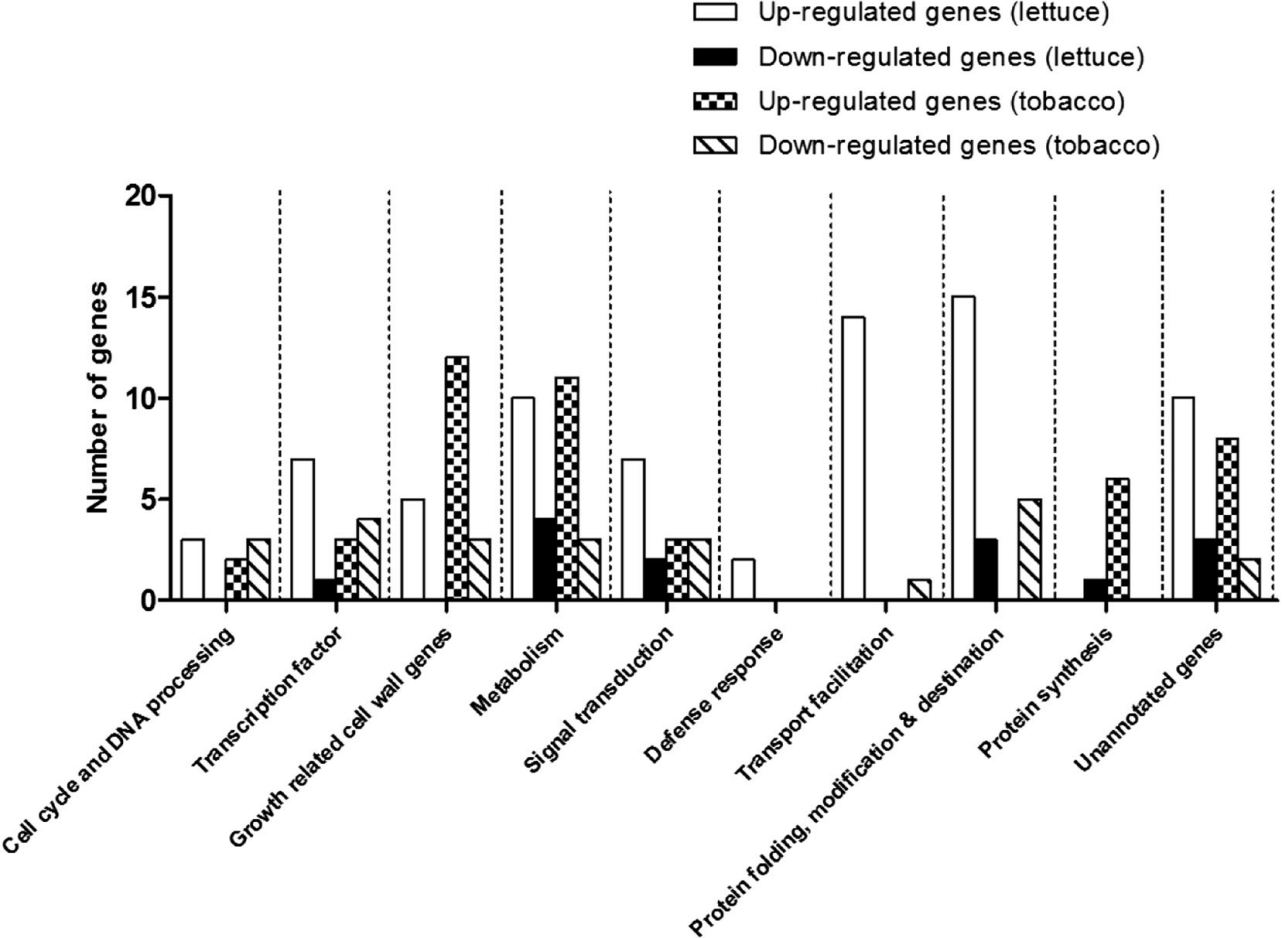
Differentially expressed genes in *CaRZFP1*-transgenic lettuce and *CaRZFP1*-transgenic tobacco plants were categorized into several groups according to their putative functions. The x-axis shows putative functional categories, and the y-axis shows the number of genes altered in expression

The expression profiles, either significantly up-regulated or significantly down-regulated, of lettuce and tobacco plants overexpressing *CaRZFP1* were dissimilar. Transgenic lettuce and tobacco did not share any genes with significantly altered expression (Table 1). And, overall expression profiles, either significantly up-regulated or significantly down-regulated, were largely different between transgenic lettuce and tobacco. In transgenic tobacco, more genes involved in protein synthesis and growth-related cell wall proteins experienced altered expression than in lettuce. However, transgenic lettuce contained more highly altered genes that were involved in transcription factors, transport facilitation, as well as protein folding, modification, and destination (Fig. 4 and Tables S1–S6).

The development of multicellular organisms depends on cellular growth and morphogenesis, involving the expression of numerous genes and intricate gene regulatory networks. RING zinc finger proteins have been closely and repeatedly implicated in the development of multiple organisms (Laity et al. 2001; Krishna et al. 2003; Gamsjaeger et al. 2007; Zhang et al. 2013), but cases reported in plants are comparatively few (Chai et al. 2015; Larrieu and Vernoux 2009; Liu et al. 2016). Enhanced growth and tolerance to abiotic stresses were observed in *CaRZFP1*-transgenic tobacco (Zeba et al. 2009), but *CaRZFP1*-transgenic lettuce displayed impaired growth (weakened leaf growth, stunted root growth, shorter plant height, and delayed flowering) compared with vector-only plants. This poor growth was strongly correlated with *CaRZFP1* expression levels. When the poor growth phenotype was investigated at the tissue and cellular level, *CaRZFP1* expression led to specific effects in lettuce. Although overall height of transgenic lettuce decreased parallel to *CaRZFP1* expression, the development of shoot internal structure was not significantly hampered. Shoot and leaf cross sections also exhibited normal development overall, as did vascular bundles, mesophyll cells, and epidermal cells. However, inflorescence development was dramatically affected by *CaRZFP1* expression, with overall size decreasing with increasing *CaRZFP1* expression. Yet transgenic flowers did not differ from control plants, although flower number per inflorescence was drastically reduced. Likewise, while transgenic seeds exhibited normal morphology, their total number per inflorescence decreased drastically with increasing *CaRZFP1* expression. Transgenic lettuce roots were strongly affected by *CaRZFP1* expression, increasing in disorganization of vascular bundles as *CaRZFP1* expression levels rose. Moreover, the xylem ray became irregular and disconnected, while the endodermis and pericycle were ill-defined and eventually unidentifiable. However, cells in root ground tissue were unaffected. Further, transgenic root length was drastically shortened, but root diameter was not significantly changed. Overall root size, mass, and branching in transgenic lettuce were reduced considerably with rising *CaRZFP1* expression levels.

In-depth studies are necessary to clearly understand the basis of the described differences in *CaRZFP1*-transgenic lettuce and tobacco. However, several possible explanations can be proposed. Because tobacco is closely related to hot pepper (both Solanaceae), hot pepper-derived CaRZFP1 was likely expressed in a similar genetic environment when mobilized to tobacco. In contrast, CaRZFP1 in lettuce was probably expressed in a very different genetic environment, likely even interacting with proteins that are not normal counterparts or binding at a DNA domain that is extremely dissimilar from any in hot pepper or tobacco. These new interactions probably caused the expression of unexpected downstream genes. Because signal transduction pathways are complex and extensively interconnected (Larrieu and Vernoux 2009), unpredictable outcomes may result once novel interactions trigger a different network of signal transductions or gene sets.

This study provides important insight into the function of C3HC4-type RING finger proteins in lettuce. We provided candidate genes downstream of the ectopically expressed *CaRZFP1* in lettuce that specifically affected root development, especially of the endodermis, pericycle, and vascular structures. We also provided strong evidence that the same gene can yield completely different outcomes depending on its host species. These results are significant because unexpected outcomes of gene mobilization to improve crops are a major safety concern. In addition to the extreme cares to control the unwanted and uncontrolled spread of transgenes between closely related wild species and the newly introduced transgenic crops through pollen diffusion, the results in this report asks for another level of concerns due to the chance of occurring unexpected phenotype appearance from the gene transfer. Likewise, the concern can be extended to the general molecular approach to decipher the function of a genetic component. Scientific researches heavily rely on a model system, for an example Arabidopsis, to decipher the function of a gene/protein, pathways, and networks in plants. If different plant species carry quite different scaffolding network for a protein or gene, the outcome can be quite different. Since plasticity of development is one important nature in plants, we tempt to see much heavier emphasis on each plant species as a host for the common and for the specificity.

## Electronic supplementary material

Table S1 (DOCX 42 kb)

Table S2 (DOCX 28 kb)

Table S3 (DOCX 22 kb)

Table S4 (DOCX 19 kb)

Table S5 (DOCX 22 kb)

Table S6 (DOCX 21 kb)

Figures S1-S3 (DOC 3.88 MB)
