# Supplementary material for: Ectopic RING zinc finger gene from hot pepper induces totally different genes in lettuce and tobacco
Source: Mol Breed. 2018 May 16;38(6):70. doi: 10.1007/s11032-018-0812-3 (PMC5956013; doi:10.1007/s11032-018-0812-3)
Supplement: Supplementary file 1 — (DOCX 42 kb) [file 11032_2018_812_MOESM1_ESM.docx]

**Table S1.** Genes up-regulated in *CaRZFP1*-overexpressing T_4_ generation lettuce plants.

| **Agilent probe set ID** | **Gene symbol** | **Gene description** | **Expression level** | | | | | ***CaRZFP1*-transgenic lettuce lines/vector controls (log_2_ fold change)** | | | | ***p*-value** |
| --- | --- | --- | --- | --- | --- | --- | --- | --- | --- | --- | --- | --- |
|  |  |  | **Average of vector control lines** | **#6** | **#14** | **#16** | **#12** | **#6** | **#14** | **#16** | **#12** |  |
| *Cell cycle and DNA processing* | | | | | | | | | | | | |
| **A_84_P11226** | **At5g53170** | **Cell division protease ftsH-11 (FTSH11)** | **2.13** | **2.60** | **2.78** | **5.49** | **11.02** | **0.28** | **0.38** | **1.36** | **2.36** | **7.40E-03** |
| **A_84_P833412** | **At5g52910** | **Timeless family protein (ATIM)** | **2.13** | **2.13** | **3.06** | **2.87** | **21.83** | **0** | **0.52** | **0.42** | **3.35** | **1.67E-02** |
| A_84_P11445 | At1g08260 | DNA polymerase epsilon subunit 1 (TIL1) | 2.13 | 2.13 | 2.13 | 2.13 | 21.78 | 0 | 0 | 0 | 3.35 | 6.40E-03 |
| *Transcription factor* | | | | | | | | | | | | |
| A_84_P824828 | At1g80490 | Topless-related protein 1 (TPR1) | 4.38 | 6.20 | 7.40 | 5.66 | 43.56 | 0.50 | 0.75 | 0.37 | 3.31 | 2.50E-02 |
| **A_84_P62920** | [**At1g55110**](https://www.arabidopsis.org/servlets/TairObject?id=137770&type=locus) | **Indeterminate-domain 7 protein (IDD7)** | **2.64** | **2.70** | **6.66** | **4.49** | **22.56** | **0.03** | **1.33** | **0.76** | **3.09** | **9.50E-02** |
| **A_84_P16708** | **At4g31920** | **Arabidopsis response regulator 10 (ARR10)** | **2.15** | **2.16** | **2.65** | **2.33** | **21.49** | **0.00** | **0.30** | **0.11** | **3.31** | **7.60E-03** |
| **A_84_P19621** | **At5g08130** | **BES1-interacting MYC-like 1 (BIM1)** | **2.13** | **2.13** | **2.45** | **2.42** | **18.06** | **0** | **0.20** | **0.18** | **3.08** | **4.80E-03** |
| A_84_P18116 | At1g80580 | Ethylene-responsive factor (ERF) | 2.13 | 2.13 | 2.13 | 2.13 | 42.73 | 0 | 0 | 0 | 4.32 | 1.30E-03 |
| A_84_P751359 | At1g08465 | Putative axial regulator YABBY 2 (YAB2) | 2.13 | 2.13 | 2.13 | 2.13 | 134.4 | 0 | 0 | 0 | 5.97 | 2.00E-04 |
| A_84_P854322 | At1g20910 | ARID/BRIGHT DNA-binding domain-containing protein | 2.13 | 2.13 | 2.13 | 2.13 | 44.69 | 0 | 0 | 0 | 4.38 | 3.80E-03 |
| *Growth related cell wall protein genes* | | | | | | | | | | | | |
| **A_84_P226209** | **At3g44370** | **Membrane insertion protein, OxaA/YidC with tetratricopeptide repeat domain-containing protein** | **2.12** | **2.46** | **4.99** | **4.28** | **13.66** | **0.21** | **1.23** | **1.01** | **2.68** | **1.59E-02** |
| **A_84_P786204** | **At1g21310** | **Extensin 3 (EXT3)** | **2.13** | **3.55** | **5.79** | **4.35** | **24.22** | **0.73** | **1.44** | **1.02** | **3.50** | **1.25E-02** |
| A_84_P829356 | At4g33610 | Glycine-rich protein | 2.13 | 2.13 | 2.13 | 2.13 | 18.07 | 0 | 0 | 0 | 3.08 | 4.40E-03 |
| A_84_P594615 | At3g28550 | Proline-rich extensin-like family protein | 2.13 | 2.13 | 2.13 | 2.13 | 17.64 | 0 | 0 | 0 | 3.04 | 7.50E-03 |
| A_84_P568734 | At3g24860 | Hydroxyproline-rich glycoprotein family protein | 2.13 | 2.13 | 2.13 | 2.13 | 16.53 | 0 | 0 | 0 | 2.95 | 4.10E-03 |
| *Metabolism* | | | | | | | | | | | | |
| **A_84_P857567** | **At4g30170** | **Peroxidase 45** | **2.13** | **2.33** | **5.40** | **3.80** | **13.43** | **0.12** | **1.34** | **0.83** | **2.65** | **1.27E-02** |
| **A_84_P20665** | **At5g48300** | **Glucose-1-phosphate adenylyltransferase small subunit (ADG1)** | **2.12** | **2.15** | **2.54** | **2.82** | **14.70** | **0.02** | **0.26** | **0.41** | **2.79** | **6.10E-03** |
| **A_84_P813705** | **At5g08530** | **NADH dehydrogenase (ubiquinone) flavoprotein 1(CI51)** | **2.13** | **2.16** | **4.97** | **6.17** | **17.29** | **0.02** | **1.22** | **1.53** | **3.02** | **1.06E-02** |
| **A_84_P258550** | **At3g03890** | **FMN binding protein** | **2.52** | **6.64** | **7.63** | **10.71** | **21.99** | **1.39** | **1.59** | **2.08** | **3.12** | **1.20E-03** |
| A_84_P21361 | At4g10120 | Sucrose-phosphate synthase | 2.13 | 2.13 | 2.13 | 2.13 | 11.61 | 0 | 0 | 0 | 2.44 | 4.30E-03 |
| A_84_P156385 | At5g66230 | Chalcone-flavanone isomerase family protein | 2.13 | 2.13 | 2.13 | 2.13 | 43.43 | 0 | 0 | 0 | 4.34 | 1.50E-03 |
| A_84_P833808 | At1g17420 | Lipoxygenase 3 (LOX3) | 2.13 | 2.13 | 2.13 | 2.13 | 52.15 | 0 | 0 | 0 | 4.60 | 1.70E-02 |
| A_84_P15837 | At5g08100 | Isoaspartyl peptidase/L-asparaginase 1 subunit beta | 2.13 | 2.13 | 2.13 | 2.13 | 53.24 | 0 | 0 | 0 | 4.63 | 1.00E-03 |
| A_84_P790164 | At5g41080 | Glycerophosphoryl diester phosphodiesterase family protein | 2.13 | 2.13 | 2.13 | 2.13 | 36.63 | 0 | 0 | 0 | 4.10 | 3.60E-03 |
| A_84_P831419 | At1g54620 | Pectin methylesterase inhibitor superfamily protein | 2.13 | 2.13 | 2.13 | 2.13 | 42.20 | 0 | 0 | 0 | 4.30 | 5.60E-03 |
| *Signal transduction* | | | | | | | | | | | | |
| A_84_P852024 | At1g06700 | Protein kinase domain-containing protein | 2.29 | 3.25 | 3.20 | 2.39 | 38.83 | 0.50 | 0.48 | 0.06 | 4.08 | 5.00E-03 |
| **A_84_P18426** | **At3g48190** | **Serine/threonine-protein kinase (ATM)** | **2.13** | **2.13** | **6.05** | **3.88** | **14.3** | **0.00** | **1.50** | **0.86** | **2.74** | **2.92E-02** |
| A_84_P212118 | At4g04960 | L-type lectin receptor kinase VII.1 (LECRK-VII.1) | 2.13 | 2.13 | 2.13 | 2.13 | 42.65 | 0 | 0 | 0 | 4.31 | 3.00E-03 |
| A_84_P14571 | At3g20860 | NIMA-related kinase 5 (NEK5) | 2.13 | 2.13 | 2.13 | 2.13 | 11.65 | 0 | 0 | 0 | 2.44 | 1.58E-02 |
| A_84_P791547 | At3g24540 | Proline-rich extensin-like receptor kinase (PERK) | 2.13 | 2.13 | 2.13 | 2.13 | 23.59 | 0 | 0 | 0 | 3.46 | 5.10E-03 |
| A_84_P570789 | At5g11360 | Interleukin-1 receptor-associated kinase 4 protein | 2.13 | 2.13 | 2.13 | 2.13 | 21.50 | 0 | 0 | 0 | 3.33 | 7.70E-03 |
| A_84_P302670 | At5g12235 | CLAVATA3/ESR-related 22 protein (CLE22) | 2.13 | 2.13 | 2.13 | 2.13 | 34.13 | 0 | 0 | 0 | 3.99 | 8.70E-03 |
| *Defense Response* | | | | | | | | | | | | |
| **A_84_P16568** | **At3g55840** | **Hs1pro-1 protein** | **2.68** | **3.91** | **6.01** | **4.93** | **66.14** | **0.54** | **1.16** | **0.87** | **4.62** | **3.20E-03** |
| A_84_P761276 | At3g61185 | Defensin-like (DEFL) family protein | 2.13 | 2.13 | 2.13 | 2.13 | 92.58 | 0 | 0 | 0 | 5.43 | **8.00E-04** |
| *Transport facilitation* | | | | | | | | | | | | |
| **A_84_P827818** | **At4g10850** | **Nodulin MtN3-like protein** | **2.13** | **2.67** | **2.83** | **2.81** | **22.46** | **0.32** | **0.40** | **0.39** | **3.39** | **1.20E-02** |
| **A_84_P818490** | **At3g07100** | **Sec24-like transport protein (ERMO2)** | **2.13** | **3.44** | **5.17** | **9.43** | **24.02** | **0.69** | **1.27** | **2.14** | **3.49** | **3.10E-03** |
| **A_84_P11158** | **At5g27100** | **Glutamate receptor 2.1 (GLR2.1)** | **2.13** | **2.24** | **2.52** | **8.41** | **12.28** | **0.06** | **0.24** | **1.97** | **2.52** | **8.20E-02** |
| **A_84_P793674** | **At5g01990** | **Auxin efflux carrier family protein** | **5.37** | **9.83** | **14.6** | **15.03** | **26.93** | **0.87** | **1.44** | **1.48** | **2.32** | **7.80E-03** |
| A_84_P808620 | At3g26520 | Tonoplast intrinsic protein 2 (TIP2) | 2.13 | 2.13 | 2.13 | 2.13 | 15.61 | 0 | 0 | 0 | 2.86 | 4.10E-03 |
| A_84_P21005 | At2g39890 | Proline transporter 1 (PROT1) | 2.13 | 2.13 | 2.13 | 2.13 | 20.66 | 0 | 0 | 0 | 3.27 | 1.12E-02 |
| A_84_P70864 | At5g51710 | K(+) efflux antiporter 5 (KEA5) | 2.13 | 2.13 | 2.13 | 2.13 | 11.84 | 0 | 0 | 0 | 2.47 | 1.91E-02 |
| A_84_P609485 | At1g79520 | Cation efflux family protein | 2.13 | 2.13 | 2.13 | 2.13 | 11.87 | 0 | 0 | 0 | 2.47 | 4.18E-02 |
| A_84_P750326 | At3g55740 | Proline transporter 2 (PROT2) | 2.13 | 2.13 | 2.13 | 2.13 | 12.51 | 0 | 0 | 0 | 2.55 | 1.85E-02 |
| A_84_P814453 | At1g80300 | Nucleotide transporter 1 (NTT1) | 2.13 | 2.13 | 2.13 | 2.13 | 26.69 | 0 | 0 | 0 | 3.64 | 3.30E-03 |
| A_84_P18458 | At1g23910 | Polyketide cyclase/dehydrase and lipid transport superfamily protein | 2.13 | 2.13 | 2.13 | 2.13 | 58.51 | 0 | 0 | 0 | 4.77 | 8.30E-03 |
| A_84_P18546 | At4g19680 | Fe(2+) transport protein 2 (IRT2) | 2.13 | 2.13 | 2.13 | 2.13 | 35.68 | 0 | 0 | 0 | 4.06 | 6.90E-03 |
| A_84_P21721 | At5g59030 | Copper transporter 1 (COPT1) | 2.13 | 2.13 | 2.13 | 2.13 | 53.89 | 0 | 0 | 0 | 4.65 | 6.10E-03 |
| A_84_P21354 | At4g08290 | Nodulin MtN21 /EamA-like transporter family protein | 2.13 | 2.13 | 2.13 | 2.13 | 63.14 | 0 | 0 | 0 | 4.88 | 1.60E-03 |
| *Protein fate (folding, modification, destination)* | | | | | | | | | | | | |
| **A_84_P12483** | **At1g31920** | **Pentatricopeptide repeat-containing protein** | **4.09** | **4.51** | **10.7** | **10.03** | **55.64** | **0.14** | **1.39** | **1.29** | **3.76** | **4.70E-03** |
| **A_84_P833611** | **At2g17600** | **Cysteine/histidine-rich C1 domain-containing protein** | **11.43** | **15.8** | **17.5** | **16.04** | **78.29** | **0.46** | **0.62** | **0.48** | **2.77** | **3.10E-03** |
| **A_84_P17816** | **At5g45428** | **Conserved peptide upstream open reading frame 24 (CPuORF24)** | **20.54** | **29.6** | **29.9** | **31.92** | **193.3** | **0.53** | **0.54** | **0.63** | **3.23** | **1.00E-04** |
| **A_84_P12134** | **At5g42990** | **Putative ubiquitin-conjugating enzyme E2 18 (UBC18)** | **5.89** | **7.15** | **10.1** | **11.88** | **26.55** | **0.27** | **0.77** | **1.01** | **2.17** | **1.06E-02** |
| **A_84_P18897** | **At1g71410** | **Armadillo/beta-catenin-like repeats-containing protein** | **2.14** | **3.62** | **7.93** | **8.86** | **18.3** | **0.75** | **1.88** | **2.04** | **3.09** | **5.50E-03** |
| **A_84_P589334** | **At4g01640** | **F-box associated ubiquitination effector family protein** | **2.14** | **2.21** | **3.39** | **3.75** | **158.9** | **0.04** | **0.66** | **0.80** | **6.21** | **6.00E-02** |
| **A_84_P866419** | **At2g35140** | **Development and cell death domain protein (DCD)** | **2.13** | **2.13** | **2.65** | **3.95** | **24.72** | **0** | **0.31** | **0.88** | **3.53** | **1.98E-02** |
| A_84_P22756 | At1g31090 | F-box domain-containing protein | 2.13 | 2.13 | 2.13 | 5.13 | 16.75 | 0 | 0 | 1.26 | 2.97 | 3.70E-03 |
| A_84_P845356 | At4g03510 | E3 ubiquitin-protein ligase (RMA1) | 2.13 | 2.13 | 2.13 | 8.86 | 23.73 | 0 | 0 | 2.05 | 3.47 | 3.40E-03 |
| A_84_P24164 | At3g62940 | Cysteine proteinases family protein | 2.13 | 2.13 | 2.13 | 2.41 | 56.35 | 0 | 0 | 0.17 | 4.72 | 4.60E-03 |
| A_84_P760418 | At3g26805 | Aspartic protease family protein | 2.13 | 2.13 | 2.13 | 2.13 | 47.46 | 0 | 0 | 0 | 4.47 | 5.70E-03 |
| A_84_P21429 | At4g30020 | PA-domain containing subtilase family protein | 2.13 | 2.13 | 2.13 | 2.13 | 68.07 | 0 | 0 | 0 | 4.99 | 2.30E-03 |
| A_84_P162213 | At1g06630 | F-box domain-containing protein | 2.13 | 2.13 | 2.13 | 2.13 | 23.16 | 0 | 0 | 0 | 3.43 | 1.75E-02 |
| A_84_P238183 | At3g23880 | F-box/kelch-repeat protein | 2.13 | 2.13 | 2.13 | 2.13 | 27.59 | 0 | 0 | 0 | 3.69 | 1.40E-02 |
| A_84_P581169 | At3g11000 | Development and cell death domain protein (DCD) | 2.13 | 2.13 | 2.13 | 2.13 | 75.02 | 0 | 0 | 0 | 5.13 | 6.00E-04 |
| *Unannotated genes* | | | | | | | | | | | | |
| **A_84_P595309** | **At2g18970** | **Uncharacterized gene** | **2.13** | **3.79** | **8.09** | **4.14** | **22.63** | **0.83** | **1.92** | **0.95** | **3.40** | **1.23E-02** |
| **A_84_P794936** | **At5g04550** | **Uncharacterized gene** | **2.70** | **2.91** | **7.33** | **8.62** | **26.57** | **0.11** | **1.43** | **1.67** | **3.29** | **6.60E-03** |
| **A_84_P11270** | **At1g55160** | **Uncharacterized gene** | **2.20** | **3.41** | **4.82** | **7.99** | **25.63** | **0.63** | **1.13** | **1.86** | **3.54** | **8.10E-03** |
| **A_84_P756169** | **At2g11630** | **Uncharacterized gene** | **2.13** | **2.17** | **2.51** | **3.23** | **140.6** | **0.02** | **0.23** | **0.59** | **6.04** | **5.00E-04** |
| **A_84_P756837** | **At2g24755** | **Uncharacterized gene** | **2.13** | **2.13** | **2.67** | **3.04** | **67.12** | **0** | **0.32** | **0.51** | **4.97** | **3.70E-03** |
| **A_84_P766236** | **At5g26775** | **Uncharacterized gene** | **2.13** | **2.13** | **4.36** | **7.01** | **225.1** | **0** | **1.02** | **1.71** | **6.71** | **4.00E-04** |
| **A_84_P508404** | **At3g11300** | **Uncharacterized gene** | **2.13** | **2.13** | **2.13** | **4.56** | **40.86** | **0** | **0** | **1.09** | **4.25** | **1.40E-03** |
| A_84_P752460 | At1g43195 | Uncharacterized gene | 2.13 | 2.13 | 2.13 | 2.13 | 39.57 | 0 | 0 | 0 | 4.21 | 4.60E-03 |
| A_84_P513598 | At2g22122 | Uncharacterized gene | 2.13 | 2.13 | 2.13 | 2.13 | 47.61 | 0 | 0 | 0 | 4.47 | 4.30E-02 |
| A_84_P105846 | At1g63610 | Uncharacterized gene | 2.13 | 2.13 | 2.13 | 2.13 | 44.73 | 0 | 0 | 0 | 4.38 | 3.20E-02 |

Group 1 genes are in bold.
