# Supplementary material for: Ectopic RING zinc finger gene from hot pepper induces totally different genes in lettuce and tobacco
Source: Mol Breed. 2018 May 16;38(6):70. doi: 10.1007/s11032-018-0812-3 (PMC5956013; doi:10.1007/s11032-018-0812-3)
Supplement: Supplementary file 2 — (DOCX 28 kb) [file 11032_2018_812_MOESM2_ESM.docx]

**Table S2.** Genes down-regulated in *CaRZFP1*-overexpressing T_4_ lettuce plants.

| **Agilent probe set ID** | **Gene symbol** | **Gene description** | **Expression level** | | | | | ***CaRZFP1*-transgenic lettuce lines/vector controls (log_2_ fold change)** | | | | ***p*-value** |
| --- | --- | --- | --- | --- | --- | --- | --- | --- | --- | --- | --- | --- |
|  |  |  | **Average of vector control lines** | **#6** | **#14** | **#16** | **#12** | **#6** | **#14** | **#16** | **#12** |  |
| *Transcription factor* | | | | | | | | | | | | |
| A_84_P824704 | At2g47850 | Zinc finger CCCH domain-containing protein 32 | 64.18 | 34.29 | 44.06 | 47.61 | 5.18 | -0.90 | -0.54 | -0.43 | -3.62 | 1.26E-02 |
| *Metabolism* | | | | | | | | | | | | |
| **A_84_P850432** | **At4g10300** | **RmlC-like cupins super family protein** | **1632** | **902.8** | **857.9** | **503.8** | **400.2** | **-0.85** | **-0.92** | **-1.69** | **-2.02** | **4.45E-02** |
| **A_84_P723766** | **At3g55850** | **Amidohydrolase family protein (LAF3)** | **2079** | **1910** | **1810** | **1459** | **335** | **-0.12** | **-0.19** | **-0.51** | **-2.63** | **2.40E-03** |
| **A_84_P18637** | **At4g15550** | **Indole-3-acetate beta-D-glucosyltransferase (IAGLU)** | **34.19** | **32.85** | **35.78** | **31.59** | **3.19** | **-0.05** | **-0.15** | **-0.11** | **-3.41** | **1.02E-02** |
| A_84_P13059 | At5g37600 | Glutamine synthetase cytosolic isozyme 1-1 (GSR 1) | 42.68 | 13.24 | 17.14 | 11.78 | 4.57 | -1.68 | -1.31 | -1.85 | -3.22 | 6.00E-03 |
| *Signal transduction* | | | | | | | | | | | | |
| **A_84_P830423** | **At3g44200** | **Serine/threonine-protein kinase Nek5 (NEK6)** | **250** | **92.17** | **90.86** | **65.86** | **59.65** | **-1.44** | **-1.46** | **-1.92** | **-2.06** | **4.08E-02** |
| **A_84_P23660** | **At1g60800** | **NSP-interacting kinase 3 (NIK3)** | **25.15** | **10.19** | **10.02** | **8.07** | **3.21** | **-1.30** | **-1.32** | **-1.63** | **-2.96** | **7.50E-03** |
| *Protein fate (folding, modification, destination)* | | | | | | | | | | | | |
| **A_84_P95886** | **At2g47990** | **Transducin family protein/WD-40 repeat family protein** | **40.71** | **26.96** | **21.51** | **12.9** | **10.12** | **-0.59** | **-0.92** | **-1.65** | **-2.00** | **6.40E-03** |
| A_84_P13790 | At4g08950 | Phosphate-responsive 1 family protein (EXO) | 47.50 | 19.30 | 18.56 | 22.29 | 2.13 | -1.29 | -1.35 | -1.09 | -4.47 | 1.93E-02 |
| A_84_P830642 | At3g02490 | Pentatricopeptide repeat (PPR) superfamily protein | 24.5 | 9.24 | 14.56 | 13.95 | 2.83 | -1.40 | -0.75 | -0.81 | -3.11 | 8.90E-03 |
| *Protein synthesis* | | | | | | | | | | | | |
| **A_84_P829794** | **AtCG00290** | **tRNA-Ser** | **4125** | **3663** | **2464** | **2464** | **657** | **-0.17** | **-0.74** | **-.074** | **-2.64** | **5.50E-03** |
| *Unannotated genes* | | | | | | | | | | | | |
| **A_84_P737379** | **At5g37650** | **Uncharacterized gene** | **50.87** | **30.94** | **24.18** | **19.51** | **5.62** | **-0.71** | **-1.07** | **-1.38** | **-3.17** | **1.86E-02** |
| A_84_P762480 | At3g52742 | Uncharacterized gene | 15.14 | 20.19 | 7.06 | 13.85 | 3.43 | -0.41 | -1.10 | -0.12 | -2.14 | 3.81E-02 |
| A_84_P762419 | At3g15518 | Uncharacterized gene | 54.62 | 11.36 | 12.52 | 16.43 | 3.52 | -2.26 | -2.12 | -1.73 | -3.95 | 3.58E-02 |

Group 1 genes are in bold.
