# Supplementary material for: Ectopic RING zinc finger gene from hot pepper induces totally different genes in lettuce and tobacco
Source: Mol Breed. 2018 May 16;38(6):70. doi: 10.1007/s11032-018-0812-3 (PMC5956013; doi:10.1007/s11032-018-0812-3)
Supplement: Supplementary file 3 — (DOCX 22 kb) [file 11032_2018_812_MOESM3_ESM.docx]

**Table S3** Up-regulated genes in *CaRZFP1*-overexpressing T_2_ tobacco plants (Zeba et al., 2009).

| **Affymetrix probe set ID** | **Gene symbol** | **Gene description** | ***CaRZFP1*-transgenic tobacco lines / vector controls (log_2_ fold change)** | ***p*-value** |
| --- | --- | --- | --- | --- |
| *Cell cycle and DNA processing* | | | | |
| 248105_at | At5g55280 | Cell division protein ftsZ | 2.21 | 2.78E-01 |
| 256238_at | At3g12400 | Tumour susceptibility gene 101 (tsg101) family protein | 3.21 | 8.79E-02 |
| *Transcriptional factor* | | | | |
| 260305_at | At1g70490 | ADP-ribosylation factor | 6.88 | 1.63E-02 |
| 256069_at | At1g13740 | ABI five binding protein 2 | 5.80 | 5.23E-02 |
| 249468_at | At5g39650 | DUO1-activated unknown 2 (DUO2) | 2.03 | 4.13E-01 |
| *Growth related cell wall protein genes* | | | | |
| 250437_at | At5g10430 | Arabinogalactan-protein (agp4) | 4.42 | 1.17E-01 |
| 247279_at | At5g64310 | Arabinogalactan-protein (agp1) | 2.52 | 1.44E-01 |
| 253050_at | At4g37450 | Arabinogalactan-protein (agp18) | 3.50 | 1.89E-03 |
| 266552_at | At2g46330 | Arabinogalactan-protein (agp16) | 2.87 | 8.11E-02 |
|  | At5g49080 | Proline-rich extensin-like family protein | 2.18 | 4.35E-01 |
| 252253_at | At3g49300 | Proline-rich family protein | 1.78 | 4.66E-01 |
|  | At3g49305 | Hypothetical protein contains  proline-rich extensin domains | 2.17 | 1.72E-01 |
| 252971_at | At4g38770 | Proline-rich family protein (prp4) | 6.05 | 1.88E-02 |
| 255138_at | At4g08380 | Proline-rich extensin-like family protein | 2.48 | 2.06E-01 |
| 245967_at | At5g19800 | Hydroxyproline-rich glycoprotein family protein | 2.72 | 1.56E-01 |
| 263046_at | At2g05380 | Glycine-rich protein(grp3) | 3.71 | 2.39E-02 |
| 261826_at | At1g11580 | Pectin methylesterase | 2.32 | 1.65E-01 |
| *Metabolism* | | | | |
| 264474_at | At5g38410 | Ribulose bisphosphate carboxylase small chain 3b | 3.49 | 3.16E-01 |
| 256865_at | At3g23820 | NAD-dependent epimerase | 2.21 | 2.28E-01 |
| 257816_at | At3g25140 | Glycosyl transferase family 8 protein | 2.86 | 1.26E-01 |
|  | At4g15233 | ABC transporter family protein | 7.31 | 1.47E-01 |
| 264394_at | At1g11860 | Aminomethyltransferase | 2.72 | 7.98E-02 |
| 247843_at | At5g58050 | Glycerophosphoryl diester phosphodiesterase  family protein | 2.43 | 1.95E-01 |
| 254802_at | At4g13090 | Xyloglucan:xyloglucosyl transferase/xyloglucan  Endotransglycosylase | 2.27 | 1.33E-01 |
| 251174_at | At3g63200 | Patatin-like protein 9 | 4.88 | 2.43E-02 |
| 260284_at | At1g80380 | Phosphoribulokinase/uridine kinase-related | 2.51 | 2.03E-01 |
| 253090_at | At4g36360 | Beta-galactosidase | 4.55 | 3.33E-02 |
| 266716_at | At2g46820 | Curvature thylakoid 1B (CURT1B) | 3.07 | 1.97E-01 |
| *Signal transduction* | | | | |
| 260221_at | At1g74670 | Gibberellin-responsive protein | 4.46 | 4.71E-02 |
| 266867_at | At2g45770 | Signal recognition particle receptor protein/ chloroplast (ftsY) similar to cell division protein | 3.06 | 1.60E-01 |
| 265073_at | At1g55480 | Plant protein family containing a PDZ, a K-box, and a TPR motif (ZKT) | 2.61 | 1.48E-01 |
| *Protein synthesis* | | | | |
| 248655_at | At5g48760 | 60s ribosomal protein L13A (RPL13aD) | 1.89 | 5.01E-01 |
| 251487_at | At3g59760 | Cysteine synthase c/O-acetylserine (thiol)-lyase isoform c | 2.22 | 2.97E-01 |
| 264849_at | At2g17360 | 40s ribosomal protein s4 (rps4a) | 2.32 | 3.01E-01 |
| 262117_at | At1g02780 | 60s ribosomal protein | 3.31 | 2.86E-01 |
| 250256_at | At5g13650 | Elongation factor family protein | 2.38 | 1.36E-01 |
| 253333_at | At4g33510 | 3-deoxy-D-arabino-heptulosonate-7-phosphate 2 (dahp2) | 2.58 | 1.11E-01 |
| *Unannotated genes* | | | | |
| 247241_at | At5g64680 | Uncharacterized gene | 2.23 | 2.51E-01 |
| 249681_at | At5g36070 | Uncharacterized gene | 5.56 | 1.49E-02 |
|  | At3g43684 | Uncharacterized gene | 2.58 | 7.40E-02 |
| 263840_at | At2g36885 | Uncharacterized gene | 4.09 | 9.03E-02 |
|  | At2g05752 | Uncharacterized gene | 4.38 | 1.29E-01 |
| 251038_at | At5g02240 | Uncharacterized gene | 3.00 | 2.64E-01 |
| 245198_at | At1g67700 | Uncharacterized gene | 1.99 | 5.09E-01 |
| 254656_at | At4g18070 | Uncharacterized gene | 2.28 | 2.71E-01 |
