# Supplementary material for: Ectopic RING zinc finger gene from hot pepper induces totally different genes in lettuce and tobacco
Source: Mol Breed. 2018 May 16;38(6):70. doi: 10.1007/s11032-018-0812-3 (PMC5956013; doi:10.1007/s11032-018-0812-3)
Supplement: Supplementary file 4 — (DOCX 19 kb) [file 11032_2018_812_MOESM4_ESM.docx]

**Table S4.** Down-regulated genes in *CaRZFP1*-overexpressing T_2_ tobacco plants (Zeba et al., 2009).

| **Affymetrix probe set ID** | **Gene symbol** | **Gene description** | ***CaRZFP1*-transgenic tobacco liens / vector controls (log_2_ fold change)** | ***p*-value** |
| --- | --- | --- | --- | --- |
| *Cell cycle and DNA processing* | | | | |
| 264399_at | At1g61780 | Postsynaptic protein-related | -1.94 | 2.41E-01 |
| 247598_at | At5g60870 | Regulator of chromosome condensation (rcc1) family protein | -2.43 | 6.68E-02 |
| 256273_at | At3g12090 | Tetraspanin gene family (TET6) | -2.43 | 6.02E-02 |
| *Transcription factor* | | | | |
| 247125_at | At5g66070 | Zinc finger (C3HC4-type ring finger) family protein | -2.03 | 2.39E-01 |
| 263152_at | At1g54060 | Trihelix DNA binding protein family | -1.80 | 2.17E-01 |
| 255495_at | At4g02720 | Ortholog of NKAP (NF-kB activating protein) proteins | -2.22 | 1.48E-01 |
| 250974_at | At5g02820 | Brassinosteroid insensitive 5 (BIN5) | -2.55 | 2.03E-02 |
| *Growth related cell wall protein genes* | | | | |
| 256964_at | At3g13520 | Arabinogalactan-protein (agp12) | -2.36 | 1.52E-01 |
| 265511_at | At2g05540 | Glycine-rich protein | -1.69 | 7.32E-02 |
| 260840_at | At1g29050 | Trichome birefringence-like 38 | -2.25 | 2.57E-01 |
| *Metabolism* | | | | |
| 266072_at | At2g18700 | Trehalose phosphatase/synthase 11 (TPS11) | -2.40 | 1.78E-01 |
| 265926_at | At2g18600 | Rub1-conjugating enzyme | -2.23 | 1.32E-01 |
| 252395_at | At3g47950 | ATPase | -2.03 | 2.57E-01 |
| *Signal transduction* | | | | |
| 257043_at | At3g19700 | Leucine-rich repeat transmembrane protein kinase | -2.17 | 1.10E-01 |
| 247306_at | At5g63870 | Serine/threonine protein phosphatase (pp7) | -2.16 | 8.73E-02 |
| 248017_at | At5g56460 | Protein kinase | -2.86 | 2.62E-02 |
| *Transport facilitation* | | | | |
| 247632_at | At5g60460 | Sec61-beta subunit family protein | -2.78 | 2.12E-02 |
| *Protein fate (folding, modification, destination)* | | | | |
| 265960_at | At2g37470 | Histone h2b | -3.19 | 9.00E-02 |
| 253826_at | At4g27960 | Ubiquitin-conjugating enzyme 9 (ubc9) | -2.35 | 2.24E-01 |
| 258954_at | At3g01400 | Armadillo/beta-catenin repeat family protein | -2.89 | 3.11E-03 |
| 247107_at | At5g66040 | Protein with thiosulfate sulfurtransferase/rhodanese activity (STR16) | -1.91 | 1.79E-01 |
| 258979_at | At3g09440 | Heat shock protein 70 (hsp70) | -1.83 | 2.08E-01 |
| *Unannotated genes* | | | | |
| 265457_at | At2g46550 | Uncharacterized gene | -2.66 | 2.06E-01 |
| 260320_at | At1g63930 | Uncharacterized gene | -2.72 | 2.79E-01 |
