# Supplementary material for: Ectopic RING zinc finger gene from hot pepper induces totally different genes in lettuce and tobacco
Source: Mol Breed. 2018 May 16;38(6):70. doi: 10.1007/s11032-018-0812-3 (PMC5956013; doi:10.1007/s11032-018-0812-3)
Supplement: Supplementary file 5 — (DOCX 22 kb) [file 11032_2018_812_MOESM5_ESM.docx]

**Table S5.** Gene ontology (GO) of up and down regulated genes in lettuce transgenic plants.

| **GO term** | **Ontology** | **Description** | **Number in input list** | **Number in BG/Ref** | **p-value** | **FDR** |
| --- | --- | --- | --- | --- | --- | --- |
| GO:0042592 | P | homeostatic process | 6 | 542 | 0.0048 | 0.97 |
| GO:0009628 | P | response to abiotic stimulus | 9 | 2022 | 0.12 | 1 |
| GO:0019222 | P | regulation of metabolic process | 8 | 3181 | 0.7 | 1 |
| GO:0009791 | P | post-embryonic development | 5 | 1576 | 0.47 | 1 |
| GO:0044249 | P | cellular biosynthetic process | 13 | 5870 | 0.88 | 1 |
| GO:0006807 | P | nitrogen compound metabolic process | 13 | 6241 | 0.93 | 1 |
| GO:0007165 | P | signal transduction | 7 | 1965 | 0.33 | 1 |
| GO:0034645 | P | cellular macromolecule biosynthetic process | 10 | 4455 | 0.84 | 1 |
| GO:0050789 | P | regulation of biological process | 14 | 5306 | 0.67 | 1 |
| GO:0009719 | P | response to endogenous stimulus | 6 | 1732 | 0.37 | 1 |
| GO:0044267 | P | cellular protein metabolic process | 9 | 3841 | 0.78 | 1 |
| GO:0000003 | P | reproduction | 8 | 1721 | 0.12 | 1 |
| GO:0044260 | P | cellular macromolecule metabolic process | 21 | 7410 | 0.56 | 1 |
| GO:0010467 | P | gene expression | 8 | 4747 | 0.97 | 1 |
| GO:0016043 | P | cellular component organization | 5 | 2456 | 0.84 | 1 |
| GO:0007275 | P | multicellular organism development | 10 | 2811 | 0.28 | 1 |
| GO:0065007 | P | biological regulation | 20 | 6062 | 0.27 | 1 |
| GO:0065008 | P | regulation of biological quality | 8 | 1255 | 0.027 | 1 |
| GO:0010468 | P | regulation of gene expression | 6 | 2814 | 0.83 | 1 |
| GO:0032502 | P | developmental process | 11 | 3233 | 0.32 | 1 |
| GO:0032501 | P | multicellular organismal process | 12 | 3032 | 0.15 | 1 |
| GO:0048608 | P | reproductive structure development | 5 | 1265 | 0.29 | 1 |
| GO:0006139 | P | nucleobase-containing compound metabolic process | 11 | 4178 | 0.66 | 1 |
| GO:0060255 | P | regulation of macromolecule metabolic process | 8 | 2998 | 0.63 | 1 |
| GO:0006810 | P | transport | 12 | 2391 | 0.039 | 1 |
| GO:0006464 | P | cellular protein modification process | 7 | 2126 | 0.41 | 1 |
| GO:0050794 | P | regulation of cellular process | 13 | 4857 | 0.65 | 1 |
| GO:0009058 | P | biosynthetic process | 14 | 6255 | 0.88 | 1 |
| GO:0003006 | P | developmental process involved in reproduction | 6 | 1496 | 0.26 | 1 |
| GO:0006950 | P | response to stress | 11 | 3506 | 0.42 | 1 |
| GO:0009059 | P | macromolecule biosynthetic process | 10 | 4511 | 0.85 | 1 |
| GO:0008152 | P | metabolic process | 33 | 12035 | 0.66 | 1 |
| GO:0007154 | P | cell communication | 7 | 2223 | 0.45 | 1 |
| GO:0051234 | P | establishment of localization | 13 | 2406 | 0.019 | 1 |
| GO:0009056 | P | catabolic process | 6 | 1430 | 0.22 | 1 |
| GO:0051179 | P | localization | 13 | 2517 | 0.026 | 1 |
| GO:0051704 | P | multi-organism process | 5 | 1631 | 0.5 | 1 |
| GO:0044238 | P | primary metabolic process | 27 | 9890 | 0.65 | 1 |
| GO:0022414 | P | reproductive process | 8 | 1715 | 0.12 | 1 |
| GO:0019538 | P | protein metabolic process | 11 | 4289 | 0.7 | 1 |
| GO:0050896 | P | response to stimulus | 20 | 6250 | 0.32 | 1 |
| GO:0048856 | P | anatomical structure development | 11 | 3146 | 0.29 | 1 |
| GO:0043412 | P | macromolecule modification | 7 | 2532 | 0.59 | 1 |
| GO:0044237 | P | cellular metabolic process | 28 | 9879 | 0.56 | 1 |
| GO:0043170 | P | macromolecule metabolic process | 22 | 8111 | 0.65 | 1 |
| GO:0009987 | P | cellular process | 35 | 12771 | 0.67 | 1 |
| GO:0005215 | F | transporter activity | 13 | 1406 | 0.00018 | 0.0066 |
| GO:0016772 | F | transferase activity, transferring phosphorus-containing groups | 11 | 1657 | 0.0075 | 0.14 |
| GO:0016301 | F | kinase activity | 9 | 1436 | 0.022 | 0.27 |
| GO:0000166 | F | nucleotide binding | 14 | 3370 | 0.096 | 0.88 |
| GO:0016740 | F | transferase activity | 15 | 3791 | 0.12 | 0.88 |
| GO:0003676 | F | nucleic acid binding | 10 | 4558 | 0.86 | 1 |
| GO:0003824 | F | catalytic activity | 27 | 9101 | 0.45 | 1 |
| GO:0003677 | F | DNA binding | 9 | 2417 | 0.25 | 1 |
| GO:0016787 | F | hydrolase activity | 6 | 3279 | 0.92 | 1 |
| GO:0005515 | F | protein binding | 12 | 3669 | 0.35 | 1 |
| GO:0003700 | F | transcription factor activity, sequence-specific DNA binding | 7 | 1729 | 0.22 | 1 |
| GO:0005488 | F | binding | 34 | 12081 | 0.59 | 1 |
| GO:0005739 | C | mitochondrion | 11 | 3687 | 0.49 | 1 |
| GO:0043229 | C | intracellular organelle | 54 | 18517 | 0.45 | 1 |
| GO:0043227 | C | membrane-bounded organelle | 53 | 18217 | 0.46 | 1 |
| GO:0005783 | C | endoplasmic reticulum | 5 | 895 | 0.11 | 1 |
| GO:0009536 | C | plastid | 14 | 4213 | 0.31 | 1 |
| GO:0005634 | C | nucleus | 27 | 9924 | 0.66 | 1 |
| GO:0016020 | C | membrane | 28 | 8532 | 0.22 | 1 |
| GO:0005622 | C | intracellular | 59 | 20721 | 0.57 | 1 |
| GO:0005737 | C | cytoplasm | 39 | 13406 | 0.48 | 1 |
| GO:0005576 | C | extracellular region | 14 | 2954 | 0.04 | 1 |
| GO:0043226 | C | organelle | 54 | 18527 | 0.45 | 1 |
| GO:0012505 | C | endomembrane system | 6 | 2143 | 0.58 | 1 |
| GO:0005886 | C | plasma membrane | 15 | 3735 | 0.11 | 1 |
| GO:0032991 | C | macromolecular complex | 7 | 2658 | 0.64 | 1 |
| GO:0043231 | C | intracellular membrane-bounded organelle | 53 | 18205 | 0.46 | 1 |
| GO:0044464 | C | cell part | 71 | 22662 | 0.049 | 1 |
| GO:0044446 | C | intracellular organelle part | 14 | 4882 | 0.54 | 1 |
| GO:0044444 | C | cytoplasmic part | 34 | 10923 | 0.3 | 1 |
| GO:0044424 | C | intracellular part | 59 | 20693 | 0.57 | 1 |
| GO:0005623 | C | cell | 71 | 22664 | 0.049 | 1 |
| GO:0044422 | C | organelle part | 14 | 4894 | 0.54 | 1 |

P, biological process; F, molecular function; C, cellular component
