# Supplementary material for: Ectopic RING zinc finger gene from hot pepper induces totally different genes in lettuce and tobacco
Source: Mol Breed. 2018 May 16;38(6):70. doi: 10.1007/s11032-018-0812-3 (PMC5956013; doi:10.1007/s11032-018-0812-3)
Supplement: Supplementary file 6 — (DOCX 21 kb) [file 11032_2018_812_MOESM6_ESM.docx]

**Table S6.** Gene ontology (GO) of up and down regulated genes in transgenic tobacco plants.

| **GO term** | **Ontology** | **Description** | **Number in input list** | **Number in BG/Ref** | **p-value** | **FDR** |
| --- | --- | --- | --- | --- | --- | --- |
| GO:0000003 | P | reproduction | 9 | 1721 | 0.018 | 0.77 |
| GO:0009987 | P | cellular process | 39 | 12771 | 0.015 | 0.77 |
| GO:0005975 | P | carbohydrate metabolic process | 7 | 1144 | 0.017 | 0.77 |
| GO:0022414 | P | reproductive process | 9 | 1715 | 0.018 | 0.77 |
| GO:0003006 | P | developmental process involved in reproduction | 8 | 1496 | 0.023 | 0.78 |
| GO:0030154 | P | cell differentiation | 5 | 768 | 0.034 | 0.82 |
| GO:0048869 | P | cellular developmental process | 6 | 1001 | 0.029 | 0.82 |
| GO:0009628 | P | response to abiotic stimulus | 8 | 2022 | 0.097 | 1 |
| GO:0044249 | P | cellular biosynthetic process | 16 | 5870 | 0.28 | 1 |
| GO:0006807 | P | nitrogen compound metabolic process | 15 | 6241 | 0.49 | 1 |
| GO:0034645 | P | cellular macromolecule biosynthetic process | 8 | 4455 | 0.83 | 1 |
| GO:0050789 | P | regulation of biological process | 8 | 5306 | 0.94 | 1 |
| GO:0009719 | P | response to endogenous stimulus | 6 | 1732 | 0.22 | 1 |
| GO:0044267 | P | cellular protein metabolic process | 10 | 3841 | 0.4 | 1 |
| GO:0044260 | P | cellular macromolecule metabolic process | 16 | 7410 | 0.68 | 1 |
| GO:0016043 | P | cellular component organization | 10 | 2456 | 0.057 | 1 |
| GO:0007275 | P | multicellular organism development | 6 | 2811 | 0.65 | 1 |
| GO:0010467 | P | gene expression | 8 | 4747 | 0.88 | 1 |
| GO:0065007 | P | biological regulation | 8 | 6062 | 0.98 | 1 |
| GO:0032502 | P | developmental process | 12 | 3233 | 0.069 | 1 |
| GO:0032501 | P | multicellular organismal process | 9 | 3032 | 0.27 | 1 |
| GO:0006139 | P | nucleobase-containing compound metabolic process | 7 | 4178 | 0.87 | 1 |
| GO:0006464 | P | cellular protein modification process | 5 | 2126 | 0.56 | 1 |
| GO:0050794 | P | regulation of cellular process | 7 | 4857 | 0.95 | 1 |
| GO:0009058 | P | biosynthetic process | 16 | 6255 | 0.38 | 1 |
| GO:0043412 | P | macromolecule modification | 6 | 2532 | 0.54 | 1 |
| GO:0009059 | P | macromolecule biosynthetic process | 8 | 4511 | 0.84 | 1 |
| GO:0008152 | P | metabolic process | 32 | 12035 | 0.19 | 1 |
| GO:0051234 | P | establishment of localization | 5 | 2406 | 0.67 | 1 |
| GO:0009056 | P | catabolic process | 5 | 1430 | 0.24 | 1 |
| GO:0051179 | P | localization | 5 | 2517 | 0.71 | 1 |
| GO:0051704 | P | multi-organism process | 6 | 1631 | 0.18 | 1 |
| GO:0044238 | P | primary metabolic process | 25 | 9890 | 0.35 | 1 |
| GO:0019538 | P | protein metabolic process | 10 | 4289 | 0.55 | 1 |
| GO:0050896 | P | response to stimulus | 20 | 6250 | 0.074 | 1 |
| GO:0048856 | P | anatomical structure development | 10 | 3146 | 0.19 | 1 |
| GO:0006950 | P | response to stress | 12 | 3506 | 0.11 | 1 |
| GO:0044237 | P | cellular metabolic process | 30 | 9879 | 0.048 | 1 |
| GO:0043170 | P | macromolecule metabolic process | 16 | 8111 | 0.82 | 1 |
| GO:0006412 | P | translation | 5 | 1500 | 0.27 | 1 |
| GO:0016817 | F | hydrolase activity, acting on acid anhydrides | 5 | 826 | 0.044 | 0.28 |
| GO:0000166 | F | nucleotide binding | 13 | 3370 | 0.045 | 0.28 |
| GO:0016740 | F | transferase activity | 14 | 3791 | 0.052 | 0.28 |
| GO:0016787 | F | hydrolase activity | 13 | 3279 | 0.038 | 0.28 |
| GO:0017111 | F | nucleoside-triphosphatase activity | 5 | 766 | 0.033 | 0.28 |
| GO:0016818 | F | hydrolase activity, acting on acid anhydrides, in phosphorus-containing anhydrides | 5 | 817 | 0.042 | 0.28 |
| GO:0016462 | F | pyrophosphatase activity | 5 | 809 | 0.041 | 0.28 |
| GO:0003824 | F | catalytic activity | 26 | 9101 | 0.13 | 0.52 |
| GO:0016788 | F | hydrolase activity, acting on ester bonds | 5 | 1131 | 0.12 | 0.52 |
| GO:0005515 | F | protein binding | 12 | 3669 | 0.14 | 0.52 |
| GO:0003676 | F | nucleic acid binding | 7 | 4558 | 0.92 | 1 |
| GO:0005488 | F | binding | 27 | 12081 | 0.65 | 1 |
| GO:0044422 | C | organelle part | 28 | 4894 | 1.60E-06 | 0.00012 |
| GO:0044446 | C | intracellular organelle part | 28 | 4882 | 1.50E-06 | 0.00012 |
| GO:0043232 | C | intracellular non-membrane-bounded organelle | 13 | 1543 | 5.10E-05 | 0.002 |
| GO:0043228 | C | non-membrane-bounded organelle | 13 | 1543 | 5.10E-05 | 0.002 |
| GO:0009579 | C | thylakoid | 8 | 584 | 6.70E-05 | 0.0021 |
| GO:0016020 | C | membrane | 35 | 8532 | 8.60E-05 | 0.0022 |
| GO:0005773 | C | vacuole | 10 | 1359 | 0.0012 | 0.023 |
| GO:0005840 | C | ribosome | 6 | 499 | 0.0011 | 0.023 |
| GO:0005886 | C | plasma membrane | 18 | 3735 | 0.0018 | 0.031 |
| GO:0031981 | C | nuclear lumen | 7 | 824 | 0.0031 | 0.048 |
| GO:0030529 | C | intracellular ribonucleoprotein complex | 7 | 852 | 0.0037 | 0.053 |
| GO:0030312 | C | external encapsulating structure | 6 | 706 | 0.0061 | 0.074 |
| GO:0005618 | C | cell wall | 6 | 706 | 0.0061 | 0.074 |
| GO:0031974 | C | membrane-enclosed lumen | 7 | 1008 | 0.0091 | 0.078 |
| GO:0043233 | C | organelle lumen | 7 | 1008 | 0.0091 | 0.078 |
| GO:0032991 | C | macromolecular complex | 13 | 2658 | 0.0077 | 0.078 |
| GO:0070013 | C | intracellular organelle lumen | 7 | 1008 | 0.0091 | 0.078 |
| GO:0005576 | C | extracellular region | 14 | 2954 | 0.0074 | 0.078 |
| GO:0044428 | C | nuclear part | 7 | 1078 | 0.013 | 0.1 |
| GO:0009536 | C | plastid | 17 | 4213 | 0.015 | 0.11 |
| GO:0005829 | C | cytosol | 9 | 1993 | 0.041 | 0.3 |
| GO:0031975 | C | envelope | 6 | 1170 | 0.055 | 0.37 |
| GO:0005794 | C | Golgi apparatus | 6 | 1182 | 0.057 | 0.37 |
| GO:0031967 | C | organelle envelope | 6 | 1170 | 0.055 | 0.37 |
| GO:0044444 | C | cytoplasmic part | 31 | 10923 | 0.1 | 0.63 |
| GO:0012505 | C | endomembrane system | 8 | 2143 | 0.12 | 0.74 |
| GO:0043231 | C | intracellular membrane-bounded organelle | 40 | 18205 | 0.77 | 1 |
| GO:0044424 | C | intracellular part | 44 | 20693 | 0.9 | 1 |
| GO:0043229 | C | intracellular organelle | 41 | 18517 | 0.75 | 1 |
| GO:0043227 | C | membrane-bounded organelle | 40 | 18217 | 0.77 | 1 |
| GO:0043226 | C | organelle | 41 | 18527 | 0.75 | 1 |
| GO:0005634 | C | nucleus | 15 | 9924 | 0.99 | 1 |
| GO:0005739 | C | mitochondrion | 7 | 3687 | 0.77 | 1 |
| GO:0044464 | C | cell part | 55 | 22662 | 0.3 | 1 |
| GO:0005623 | C | cell | 55 | 22664 | 0.3 | 1 |
| GO:0005622 | C | intracellular | 44 | 20721 | 0.9 | 1 |
| GO:0005737 | C | cytoplasm | 35 | 13406 | 0.21 | 1 |

P, biological process; F, molecular function; C, cellular component
