# Supplementary material for: Ectopic RING zinc finger gene from hot pepper induces totally different genes in lettuce and tobacco
Source: Mol Breed. 2018 May 16;38(6):70. doi: 10.1007/s11032-018-0812-3 (PMC5956013; doi:10.1007/s11032-018-0812-3)
Supplement: Supplementary file 7 — (DOC 3.88 MB) [file 11032_2018_812_MOESM7_ESM.doc]

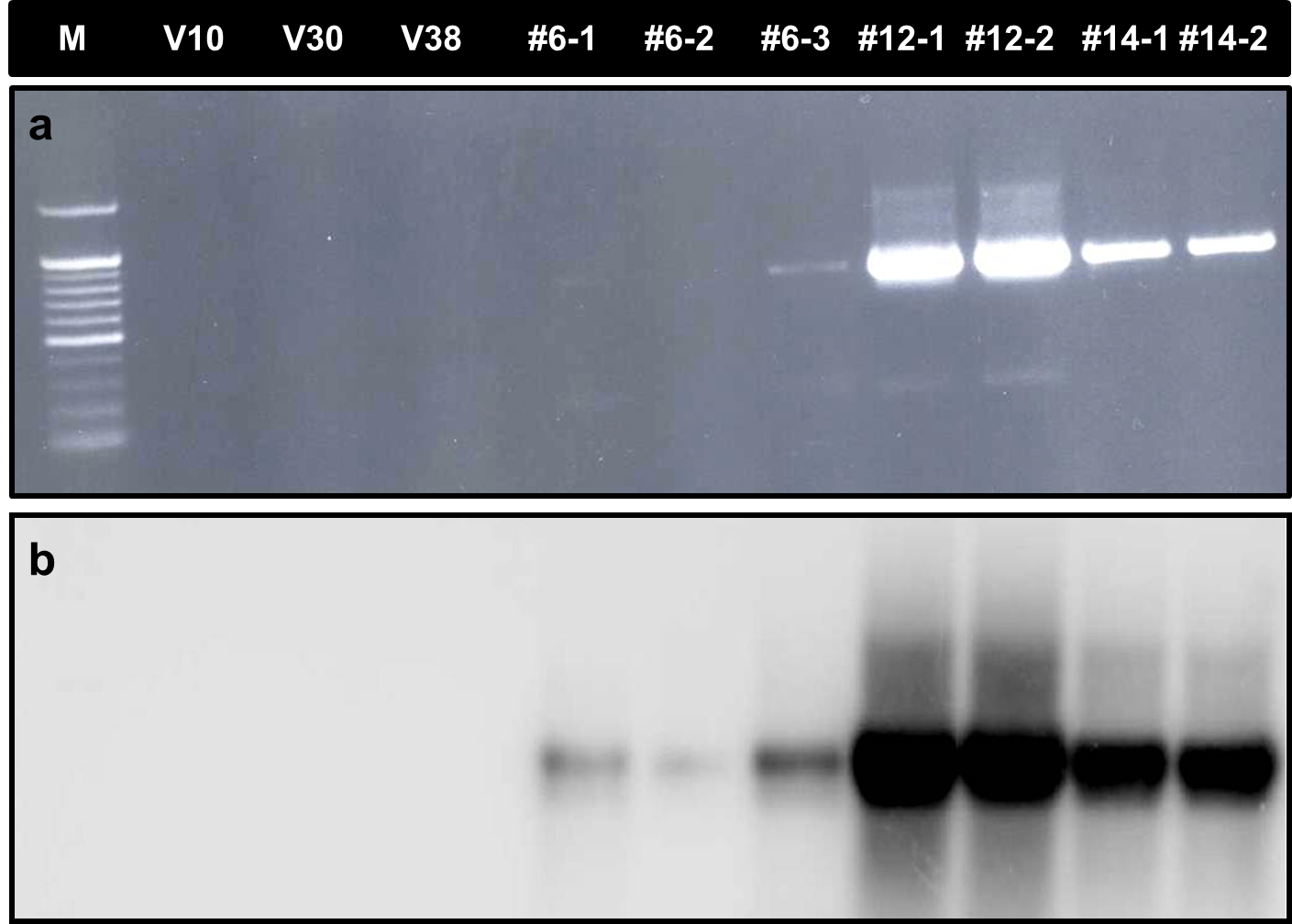


**Fig. S1** One step RT-PCR analysis for the *CaRZFP1* transcript in the *CaRZFP1*-transgenic lettuce plants. **a.** RT-PCR results with a primer pair enclosing the ORF of *CaRZFP1* for three vector-only control lines and several *CaRZFP1*-transgenic lines. **b.** RT-PCR results were further confirmed by DNA blot analysis using ^32^P-labeled *CaRZFP1* probe. M, DNA size marker.

**
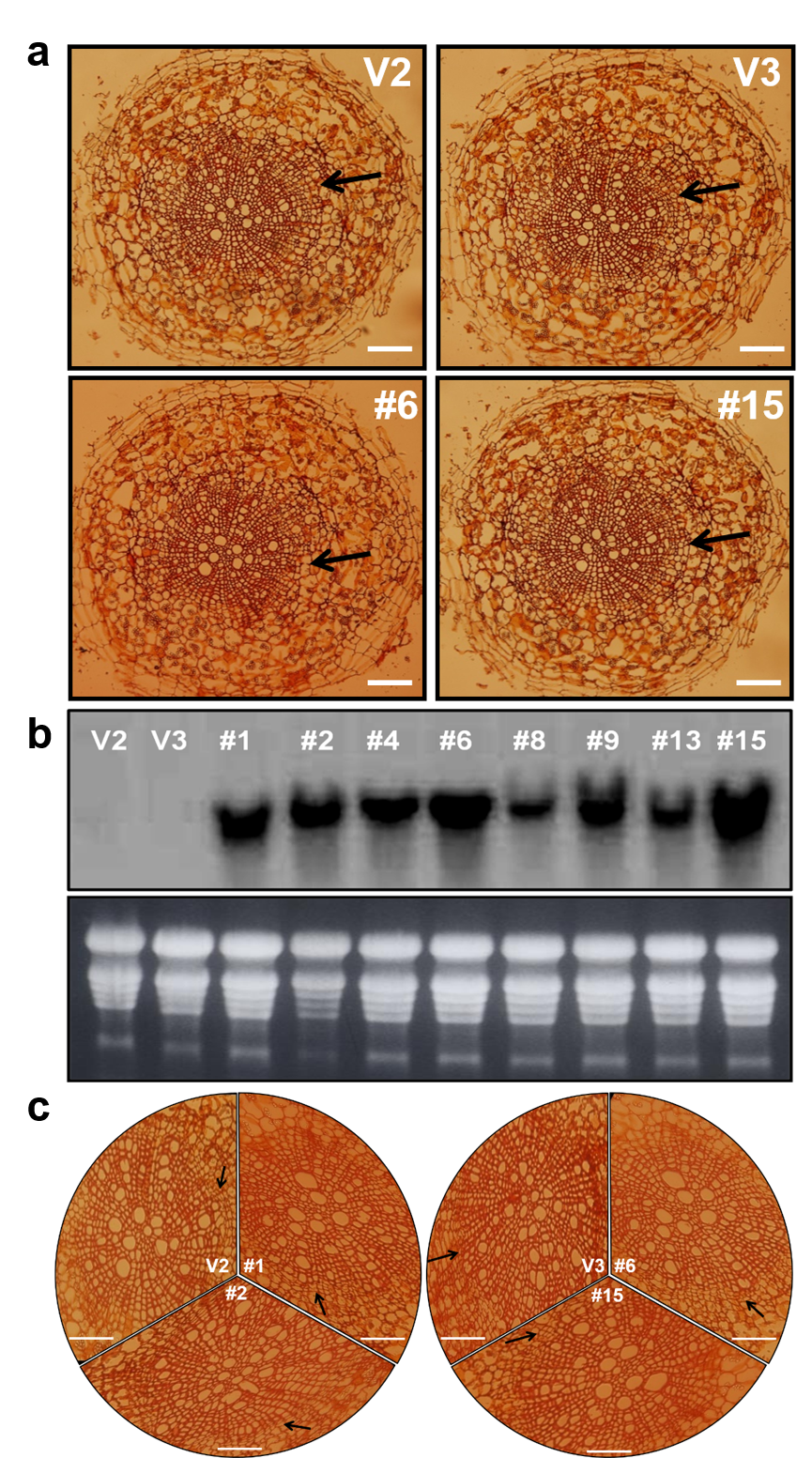
**

**Fig. S2** Roots of *CaRZFP1*-transgenic tobacco plants showed normal internal structure. **a**. Cross sections of the vector-only control lines, V2 and V3, and *CaRZFP1*-transgenic lines, #6 and #15. **b**. RNA blot hybridization results for several transgenic tobacco lines for *CaRZFP1* transcript. **c**. Magnified view of the root cross sections of the vector-only lines and the *CaRZFP1*-transgenic lines. Arrows point to the endodermis. Scale bars are for 25 µm.


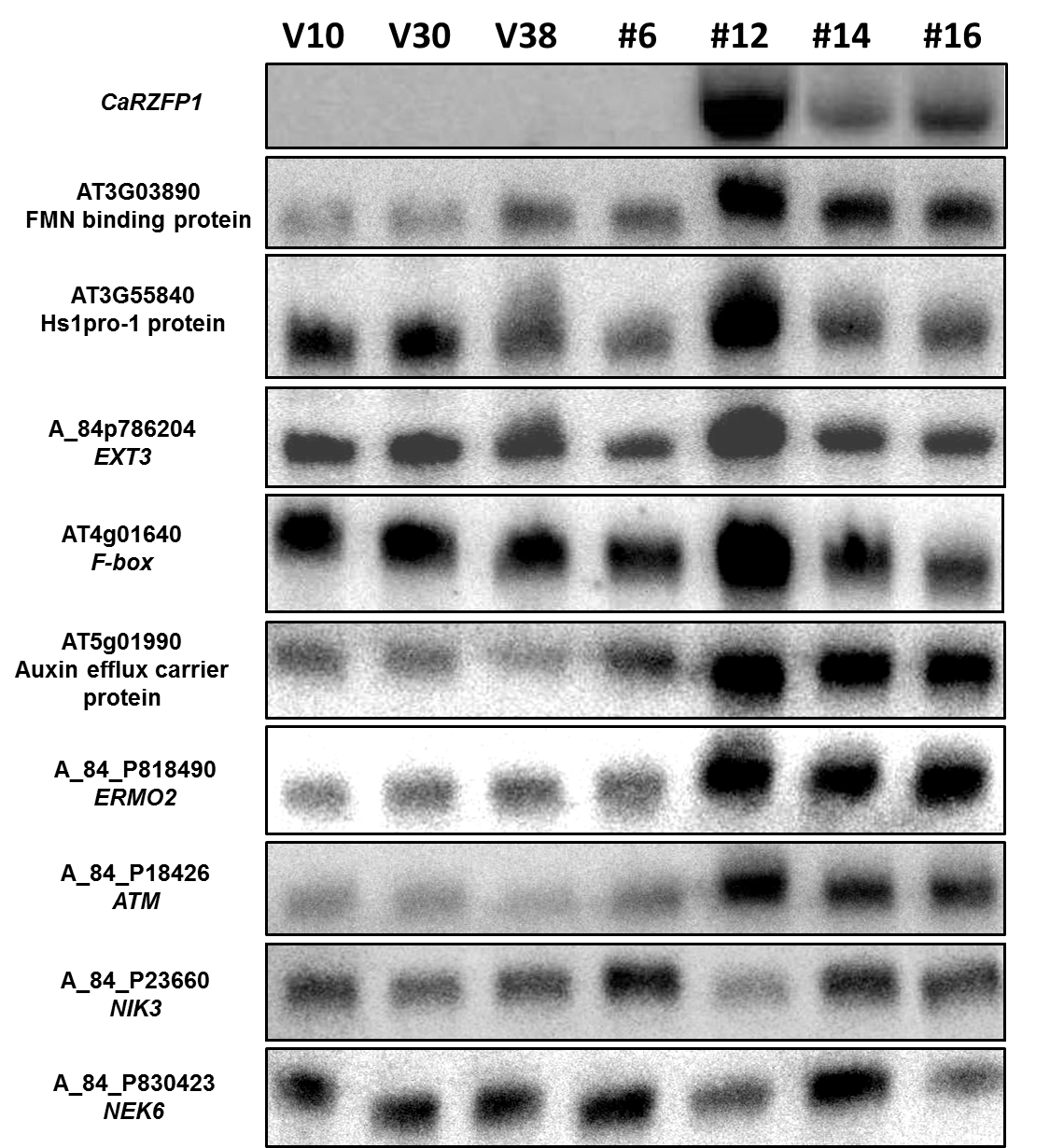


**Fig. S3** RNA blot hybridization results using oligonucleotides for the differentially expressed transcripts in the *CaRZFP1*-transgenic and vector-only lettuce plants to confirm transcriptome profile data produced by microarray analysis. Nine up- and down-regulated genes from the microarray results were randomly chosen, the genes encoded an FMN binding protein, Hs1pro-1 protein, *EXT3*, *F-box*, auxin efflux carrier protein, *ERMO2*, *ATM*, *NIK3*, and *NEK6*. Oligonucleotide sequences corresponding to the genes on the microarray array chips were labeled with ^32^P, and used for RNA blot analysis. Total RNAs from three vector-only control lines and four *CaRZFP1*-transgenic lines, #6, #12, #14, and #16, were separated by electrophoresis on an agarose gel, blotted to a membrane, and hybridized with^32^P -labeled oligonucleotides. Although some variation existed between the microarray and RNA blot results, trends in the differentially expressed genes were generally consistent across the two different approaches.
